# Genome-wide identification and comparative *in-silico* characterization of *β*-galactosidase (GH-35) in ascomycetes and its role in germ tube development of *Aspergillus fumigatus via* RNA-seq analysis

**DOI:** 10.1371/journal.pone.0286428

**Published:** 2023-06-22

**Authors:** Danish Ilyas Baig, Zeeshan Zafar, Haris Ahmed Khan, Amna Younus, Muhammad Faraz Bhatti

**Affiliations:** 1 Atta-Ur-Rahman School of Applied Biosciences, National University of Sciences and Technology, Islamabad, Pakistan; 2 National Institutes of Health (NIH), Islamabad, Pakistan; Cukurova University: Cukurova Universitesi, TURKEY

## Abstract

*β*-galactosidase (Lactase), an enzyme belonging to the glycoside hydrolase family causing the hydrolysis and trans-glycosylation of *β*-D-galactosides, has a vital role in dairy industries. The current investigation emphasizes on *in-silico* identification and comparative analysis of different fungal lactases present in *Aspergillus fumigatus*, *Aspergillus oryzae*, *Botrytis cinerea*, and *Fusarium fujikuroi*. Prediction of motifs and domains, chromosomal positioning, gene structure, gene ontology, sub-cellular localization and protein modeling were performed using different bioinformatics tools to have an insight into the structural and functional characteristics of *β*-galactosidases. Evolutionary and homology relationships were established by phylogenetic and synteny analyses. A total of 14 *β*-gal genes (GH-35) were identified in these species. Identified lactases, having 5 domains, were predicted to be stable, acidic, non-polar and extracellularly localized with roles in polysaccharide catabolic process. Results showed variable exonic/intronic ratios of the gene structures which were randomly positioned on chromosomes. Moreover, synteny blocks and close evolutionary relationships were observed between *Aspergillus fumigatus* and *Aspergillus oryzae*. Structural insights allowed the prediction of best protein models based on the higher ERRAT and Q-MEAN values. And RNA-sequencing analysis, performed on *A*. *fumigatus*, elucidated the role of *β*-gal in germ tube development. This study would pave the way for efficient fungal lactase production as it identified *β*-gal genes and predicted their various features and also it would provide a road-way to further the understanding of *A*. *fumigatus* pathogenicity via the expression insights of *β*-gal in germ tube development.

## 1. Introduction

Enzymes play a critical role in various industries ranging from food and textiles to fuels and energy generation. Microbial sources are the preferred fronts to enzymes and among them, fungal enzymes are getting more attention owing to their comparative stability and ease of extraction. *β*-galactosidase (*β*-gal) plays a vital role in the food industry especially in manipulation of dairy products for their efficient utilization [1]. *β*-galactosidase, also known as lactase enzyme, belongs to the glycoside hydrolase family that causes the hydrolysis and trans-galactosylation of *β*-D-galactosides. The common substrate for the said enzyme is lactose (having a *β*-galactosidic bond) that is either cleaved into galactose and glucose (involved in glycolysis) or converted into allolactose that ultimately converts into monosaccharides [2]. Structurally, this enzyme consists of a tetramer of 4 alike polypeptide chains (each one comprises 1023 amino acids) and five definite structural domains. The domains consist of a jelly roll barrel, fibronectin and *β*-sandwich along with a central domain of a TIM-type barrel that obliges as the active site. The catalytic site is present in central domain and it contains the subunits from all 4 chains and so the detachment of tetramer into dimers causes the inactivity of the active site. This describes the structure of a model *Escherichia coli β*-galactosidase [3, 4]. *β*-galactosidases have been classified into GH (glycosyl hydrolase) family as GH1, GH2, GH35, GH42, GH59, and GH147, in the *CAZ*y (Carbohydrate-Active enZymes) database (http://www.cazy.org/). GH2 (glycoside hydrolase 2) and GH35 (glycoside hydrolase 35) constitute the two main families of *β*-galactosidases.

Lactase deficiency causes lactose intolerance which is an abnormal condition characterized by the deficiency of lactase in the small intestine leading to the failure in digesting dairy products by the human body. Accumulation of lactose in the intestine causes flatulence, cramps, and diarrhea due to tissue dehydration and reduction in calcium absorption [5, 6]. Lactose intolerant patients are found to be at an elevated risk of developing several extra-intestinal diseases (including cancer). Its prevalence around the world ranges between 57% and 65% [7]. In Pakistan, about 60% of adults are suffering from lactose intolerance even though Pakistan holds its position among the world’s largest milk-producing countries by producing 45 billion milk annually [8].

The global annual production of *β*-galactosidase is about 5.75 million tons [9]. *β*-galactosidase has a diverse range of applications in the medical, analytical, and dairy sectors. Milk and dairy products are pre-hydrolyzed by *β*-gal to reduce the lactose concentration and make them consumable for lactose intolerant patients [10]. *β*-gal-containing medicines are also available to be taken before consuming lactose-containing products and fungal lactases are mostly used in it as they are stable at the low pH of the stomach [11, 12]. Reducing crystallization in condensed milk and ice creams and improving their texture and digestibility owe to the application of *β*-gal. In yogurts and cheese production, pH can be easily maintained by using *β*-gal and it is also used as an artificial sweetener in dairy products without altering the number of calories [13]. Hydrolysis of lactose into glucose and galactose by *β*-gal also makes them useable for the production of ethanol [14]. Whey contains minerals, proteins, and lactose and it can be converted into protein concentrates by the activity of lactase [15]. Moreover, few of these proteins can be used as pharmaceutical intermediates such as lactalbumin [13]. Conversion of whey into specific products (sweet syrups) for confectionaries and bakeries is possible by *β*-gal [16]. Galacto-oligosaccharides (GOS) produced by the trans-glycosylation of lactose by *β*-galactosidase help in the modification and growth of human intestinal microflora (probiotics) to promote human health, by acting as prebiotics [17–19]. Other than GOS, various glycosides can also be formed by *β*-galactosidase as they have broad substrate specificity [20]. *β*-galactosidase also has the potential to be used as a biosensor for the detection of lactose; it is used to detect mastitis, blood lactose levels as well as treating milk processing waste water [21].

Microbes, plants, and animals constitute the sources of *β*-galactosidase. Microbial sources (mainly *Aspergillus* and *Kluyveromyces*) are preferred due to their cost-effectiveness and higher productivity [16]. Optimum pH values of the enzyme vary with the source making their utilization specific to the desired reactions [16, 22]. Ease of fermentation, stability and high activity of *β*-galactosidase from bacterial sources make them useful for lactose hydrolysis. *Bifidobacterium* and *Lactobacillus* species are extensively used as potential sources of lactase [23]. Fungal *β*-galactosidases are more effective in the enzymatic treatment of whey (acidic substance) for lactose hydrolysis as their optimum pH mostly resides in the acidic range of 2.5–5.4. *Kluyveromyces fragilis*, *Kluyveromyces lactis* and *Aspergillus* species constitute the GRAS (generally recognized as safe) fungal sources, approved by FDA and are used on commercial scale for lactase production [16, 24]. Hence, fungi owing to their high productivity and a wide working pH range for *β*-gal could be a better option than others.

Excessive market demand for *β*-galactosidase pleas for novel/effective sources of the enzyme and this study focuses on *in-silico* identification of *β*-galactosidase in fungal species as fungal *β*-galactosidases are thermostable. These can work on a wide pH range (mostly acidic) and are known to be the best commercial sources of *β*-galactosidase [24]. Besides, structural and genetic insights into the commercially important enzyme are of great concern.

In current study, identification of *β*-galactosidase genes (GH-35) in fungal species of *Aspergillus oryzae*, *Aspergillus fumigatus*, *Botrytis cinerea*, and *Fusarium fujikuroi* along with their *in-silico* characterization and comparative analyses was performed. This investigation provides an inclusive insight into the structural and functional roles of fungal *β*-gal genes. Additionally, functional role of *β*-galactosidase in germ tube development of *Aspergillus fumigatus* was discussed to elucidate its importance in pathogenicity of fungi. The exposure to nutritious conditions allows the *Aspergillus fumigatus* conidia to set free from dormancy and develop into a swollen state (isotropic growth stage), followed by the unidirectional growth (polarized growth) that eventually leads to the tubal growth, i.e., the germ tube. Germ tube has a significant role in the fungal pathogenesis as it further differentiates and develops into hyphae [25]. *A*. *fumigatus* germ tube stimulates the immune signaling more vigorously as compared to its conidia, leading to the invasive aspergillosis (an acute lung disease) [26, 27]. So, a careful consideration regarding germ tube development would be essential to tackle the fungal pathogenicity.

## 2. Materials and methods

### 2.1 Identification of *β*-galactosidase genes from 4 fungal species

Protein FASTA files of the fungi were retrieved from the latest genome assembly available on NCBI (https://www.ncbi.nlm.nih.gov/, accessed on 20 March, 2022)-(GCF_000002655.1 for *A*. *fumigatus*, GCF_000184455.2 for *A*. *oryzae*, GCF_000143535.2 for *B*. *cinerea* & GCF_900079805.1 for *F*. *fujikuroi*) to be used as a local database to perform BLASTp. Previously reported 10 protein sequences of the *β*-gal gene from other fungal species (*Aspergillus piperis*_RAH52028.1, *Aspergillus avenaceus*_ KAE8153091.1, *Aspergillus fischeri*_EAW22192.1, *Ascochyta rabiei*_KZM26721.1, *Pterula gracilis*_TFK95762.1, *Rhizoctonia solani*_EUC61741.1, *Fusarium oxysporum*_EXK29145.1, *Pseudozyma hubeiensis*_GAC98695.1, *Penicillium digitatum*_XP_014530847.1 *& Neurospora crassa_*EAA36546.3) were retrieved from NCBI (https://www.ncbi.nlm.nih.gov/, accessed on 20 March, 2022) to identify *β*-gal genes in the selected fungal strains. Local BLASTp was performed with these retrieved sequences of *β*-gal as a query *via* BIOEDIT tool with BLOSUM62 matrix and 10 as E-value. All BLAST hits were checked for the presence of *β*-gal proteins by using the Pfam server (https://pfam.xfam.org/, accessed on 20 March, 2022). Identified *β*-gal genes were renamed based on their position on the chromosome.

### 2.2 Multiple sequence alignment and phylogenetic analysis of *β*-gal

Protein sequences of *β*-gal genes of all four fungal strains were aligned using FASTA files along with some annotated protein sequences of other fungal species (renamed as NcrBG for *Neurospora crassa*, AfiBG for *Aspergillus fischeri*, PdiBG for *Penicillium digitatum*, FoxBG for *Fusarium oxysporum* & RsoBG for *Rhizoctonia solani β*-galactosidases) and ClustalW program was used for multiple sequence alignment by using MEGA-X software (https://www.megasoftware.net/, accessed on 25 March, 2022). For phylogenetic tree construction, the output file of multiple sequence alignment was utilized using the Neighbor-joining (NJ) method with p-distance and 10,000 bootstraps replicate. Visualization and refining of the phylogenetic tree were carried out using the iTOL online webserver (https://itol.embl.de/, accessed on 3 April, 2022).

### 2.3 Chromosomal distribution of *β*-gal genes

The position of all *β*-gal genes of under-studied fungi on their respective chromosomes was examined *via* the Phenogram webserver (http://visualization.ritchielab.org/phenograms/plot, accessed on 12 April, 2022).

### 2.4 Domain & motif analysis and gene structure of *β*-gal genes

Domains of *β*-gal proteins were analyzed in NCBI conserved domain database (CDD) (https://www.ncbi.nlm.nih.gov/Structure/bwrpsb/bwrpsb.cgi, accessed on 17 April, 2022). CDD batch table was downloaded and used in TBTOOLS [28] to generate a domain graph. MEME web server (https://meme-suite.org/meme/, accessed on 17 April, 2022) was used to identify Motifs in the protein sequences of *β*-gal. Gene Structure Display Server (http://gsds.gao-lab.org/, accessed on 26 April, 2022) was used to predict the gene structure of *β*-gal genes by using gene sequences and CDS of these genes.

### 2.5 Synteny analysis of *β*-gal genes

Genomic FASTA files and genomic feature files (GFF) of *B*. *cinerea*, *A*. *fumigatus*, *A*. *oryzae & F*. *fujikuroi*, downloaded from the NCBI database, were used as input files in “One step MCScanX” of TBTools. Then syntenic blocks were visualized by placing output files of one step MCScanX in “Dual synteny plot for MCScanX” in TBTools.

### 2.6 Physicochemical properties and sub-cellular localization prediction

Molecular weight, pI, aliphatic and GRAVY index of identified proteins of *β*-gal were evaluated by predicting their physicochemical properties, using the ProtParam-EXPASY webserver (https://web.expasy.org/protparam/, accessed on 29 March, 2022). The subcellular localization of enzymes was predicted by using CELLO software (http://cello.life.nctu.edu.tw/ accessed on 2 May, 2022) and further validated by the WoLF PSORT server (https://wolfpsort.hgc.jp/, accessed on 2 May, 2022). Amino acid composition and functional motifs of proteins aid WoLF PSORT to predict localization.

### 2.7 Gene ontology of *β*-gal genes

Gene ontology was performed *via* Blast2GO tool (https://www.blast2go.com/, accessed on 14 July, 2022) to predict biological processes and molecular functions of *β*-gal genes. BLASTx was performed by using CDS sequences of *β*-gal genes, followed by InterPro Scan, BLAST2GO Mapping, and BLAST2GO Annotation under default settings of Blast2GO.

### 2.8 Signal peptide prediction of identified fungal *β*-gal

SignalP 5.0 webserver (https://services.healthtech.dtu.dk/service.php?SignalP-5.0, accessed on 17 May, 2022) was used to predict signal peptides of identified proteins of fungi by using FASTA sequences of proteins.

### 2.9 Structure prediction of *β*-gal

Tertiary structure prediction of *β*-gal enzymes was done by using the Robetta webserver (https://robetta.bakerlab.org/, accessed on 22 May, 2022). After the removal of signal peptide sequences, enzyme sequences were used for structure prediction. RoseTTAFold server of Robetta was used as it is one of the most reliable structure predictors. RoseTTAFold is based on a “3-track neural network” as it considers the patterns in sequences, the interaction of amino acids, and a possible 3D structure of protein [29]. RoseTTAFold server predicted 5 models of each submitted *β*-gal protein sequence. These models were then evaluated to select the best ones based on ERRAT values and Ramachandran plots (*via* PROCHECK) from the SAVES v6.0 UCLA server (https://saves.mbi.ucla.edu/, accessed on 25 May, 2022) and Q-mean values from the QMEAN-SWISS MODEL server (https://swissmodel.expasy.org/qmean/, accessed on 25 May, 2022). Then, a best protein model for each species was selected. The best models were refined by 3D refine webserver (http://sysbio.rnet.missouri.edu/3Drefine/, accessed on 29 May, 2022).

Binding sites of the refined protein models were predicted using DoGSiteScorer webserver (https://bio.tools/dogsitescorer, accessed on 9 June, 2022). Information about catalytic site’s active residues is specified through binding site prediction. Superimposition of refined protein models was performed in PyMOL (https://pymol.org/2/) to check the similarity with the known *β*-gal protein structure of *Aspergillus oryzae* (4IUG) from RCSB PDB (https://www.rcsb.org/structure/4IUG).

### 2.10 Differential expression analysis of *β*-gal genes of *A*. *fumigatus*

NCBI GEO database (https://www.ncbi.nlm.nih.gov/geo/query/acc.cgi?acc=GSE152682, accessed on 30 April, 2022) was used to retrieve RNA sequencing data of *A*. *fumigatus* CEA17_ΔakuBKU80 (Illumina HiSeq 2500). The maintenance of the strain was carried out at ambient temperature on 2% malt-agar slants. Transcriptome data of three replicates each on 2 media (GPA and MM) was obtained by [30] to assess the comparison in germinated conidial samples. GPA medium consisted of D-glucose 10g/L with KH_2_PO_4_ 1.52 g/L and ammonium tartrate dibasic 0.92 g/L and MM (minimal medium) consisted of D-glucose 10 g/L, KH_2_PO_4_ 1.52 g/L, ammonium tartrate dibasic 0.92 g/L, KCl 0.52 g/L, MgSO_4_ 0.52 g/L and trace element solution (Na_2_B_4_O_7_, CuSO_4_, MnSO_4_, Na_2_MoO_4_, ZnSO_4_). 34.5 g/L of morpholinepropanesulfonic acid was used in both media to avoid pH change. For the synthesis of all macromolecules of fungi, essential macronutrients are glucose, ammonium and phosphate and so are required for *A*. *fumigatus* growth. This study was designed to know the effects of a pre-defined MM medium and the GPA medium during the germination of *A*. *fumigatus*. And as we know that enzyme (*β*-gal) production varies with the ingredients of a culture media, so a link could be established between *β*-gal and the fungal germination mode. Transcriptome data was taken on the same morphological appearances in both media, ensured by 30h incubation of *A*. *fumigatus* in GPA medium and 11h incubation in MM medium.

UseGalaxy web server (https://usegalaxy.eu/, accessed on 5 May, 2022) was used to perform the analytical procedure of differential expression analysis. Sequence read archives (SRA) files were uploaded and converted into FASTQ files. FASTQC was used to assess the quality of reads at each step. Cutadapt was used to remove low-quality sequences and adapter sequences from the data, with the minimum length and quality cut-off value being set to 20 nt. RNA STAR was used to align reads with the reference genome of *A*. *fumigatus* (GCF_000002655.1). Reads per gene frequency were determined by using FeatureCounts. DESeq2 tool was used to normalize and exhibit differential gene expression. Filtration of insignificantly expressed genes was carried out by limiting the adjusted *p*-value to 0.05 and absolute fold change, FC > 2. Differentially expressed genes were visualized by creating volcano plots *via* DESeq2 data. Finally, differentially expressed *β*-gal genes were highlighted in volcano plots.

## 3. Results

### 3.1 Identification of *β*-galactosidase in *Botrytis cinerea*, *Aspergillus fumigatus*, *Aspergillus oryzae & Fusarium fujikuroi*

A total of 14 *β*-gal genes were identified in all four fungal strains based on enzyme-specific domains (four each in *B*. *cinerea*, *A*. *fumigatus*, and *A*. *oryzae* and two in *F*. *fujikuroi*). The *β*-gal genes of *B*. *cinerea* and *A*. *oryzae* were not previously reported. For the sake of convenience, these genes are renamed based on the species name (first 3 letters), protein name (next 2 letters), and chromosomal order (last digit) as BciBG1 to BciBG4 for *B*. *cinerea*, AfuBG1 to AfuBG4 for *A*. *fumigatus*, AorBG1 to AorBG4 for *A*. *oryzae* and FfuBG1, FfuBG2 for *F*. *fujikuroi* respectively. The protein sequence length of *β*-gal genes ranged from 983 amino acids of AfuBG4 to 1024aa of AfuBG2. Identified *β*-gal genes, along with their various attributes, are given in S1 File.

### 3.2 Multiple sequence alignment and phylogenetic analysis

**Fig 1:**
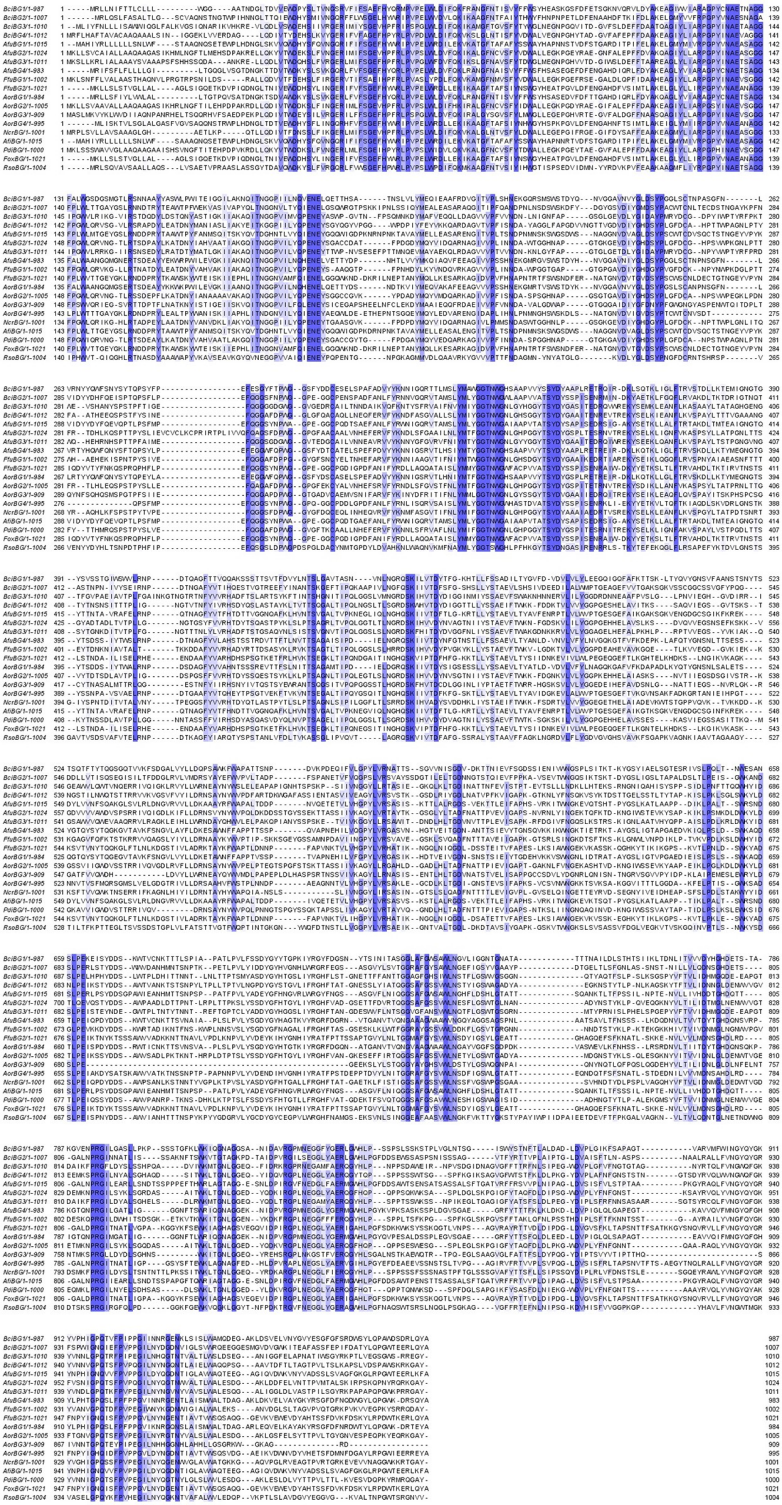
Multiple sequence alignment of identified *β*-galactosidase sequences with some annotated *β*-galactosidase sequences of *Neurospora crassa*, *Aspergillus fischeri*, *Penicillium digitatum*, *Fusarium oxysporum* and *Rhizoctonia solani*. Conserved sequences of the enzymes are highlighted. Conservancy increases from light blue to dark blue color.

**Fig 2:**
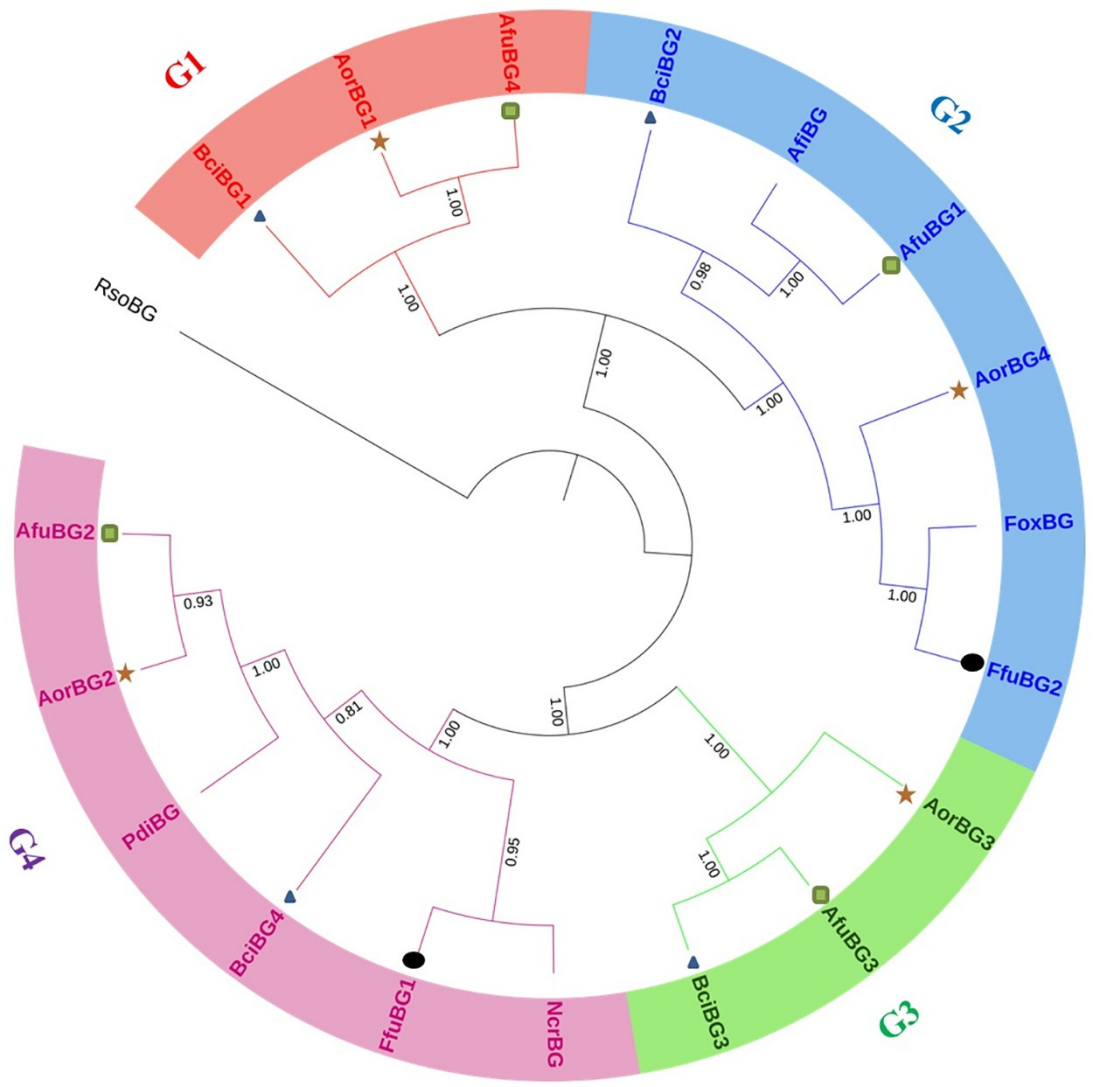
Phylogenetic tree of identified *β*-galactosidase sequences with some annotated *β*-galactosidase sequences of *Neurospora crassa*, *Aspergillus fischeri*, *Penicillium digitatum*, *Fusarium oxysporum* with *Rhizoctonia solani* as its out-group, through NJ method having 10,000 bootstrap values. It is divided into four groups (G1, G2, G3 and G4).

Multiple sequence alignment performed using MEGA-X showed significant alignment results with similar domain patterns and conserved amino acids are visible as can be seen in Fig 1. A phylogenetic tree constructed using the Neighbor-joining method had been divided into 4 groups (clades distribution) with *Rhizoctonia solani β*-gal (RsoBG) as its out-group. Group 1 (G-1) included BciBG1, AfuBG4, and AorBG1. Group 2 (G-2) included AfuBG1, AfiBG, BciBG2, AorBG4, FfuBG2, and FoxBG. Group 3 (G-3) included BciBG3, AfuBG3, and AorBG3. Group 4 (G-4) included FfuBG1, NcrBG, BciBG4, PdiBG, AfuBG2, and AorBG2. One of the *F*. *fujikuroi β*-gal (FfuBG2) was directly linked to the *F*. *oxysporum β*-gal while another one (FfuBG1) was directly linked to the *N*. *crassa β*-gal. *A*. *oryzae* and *A*. *fumigatus β*-gals (AorBG2, AfuBG2) were related to *P*. *digitatum β*-gal while one of the *A*. *fumigatus* (AfuBG1) *β*-gal was sharing a clade with *A*. *fischeri* one. *B*. *cinerea β*-galactosidases are distantly related to all other enzymes except for one enzyme of *A*. *fumigatus* (BciBG3 & AfuBG3). *A*. *oryzae* enzymes were closely related to *A*. *fumigatus* enzymes as compared to others. Phylogenetic analysis is shown in Fig 2.

### 3.3 Chromosomal distribution of *β*-gal genes

**Fig 3:**
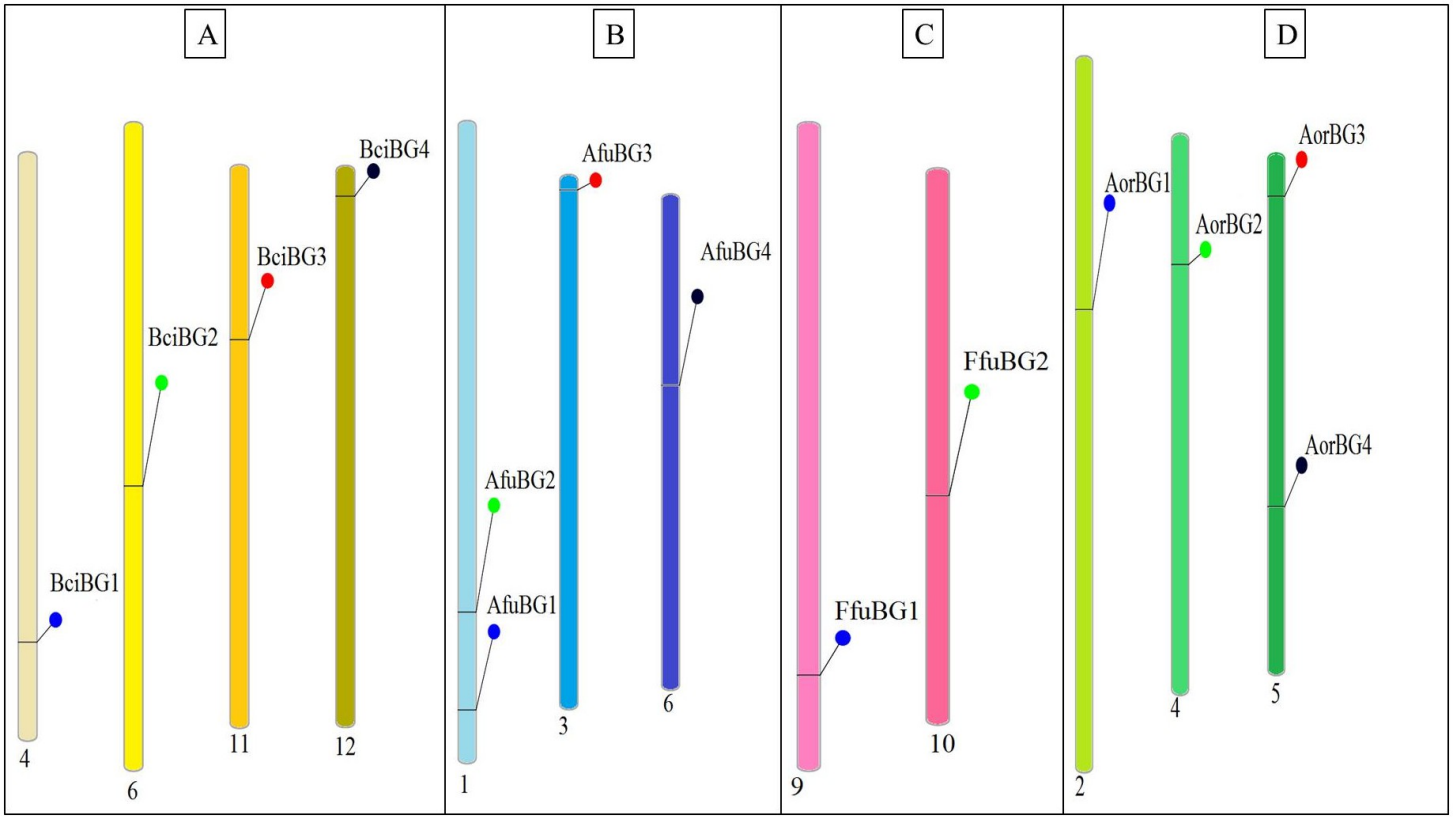
Chromosomal distribution of *β*-gal genes on chromosomes of (A) *B*. *cinerea*, (B) *A*. *fumigatus*, (C) *F*. *fujikuroi* & (D) *A*. *oryzae*. Chromosome number is mentioned below each chromosome. Different color patterns are given to each species’ chromosomes.

Chromosomal positioning predicted by Phenogram indicated that out of 18 chromosomes, four of the *B*. *cinerea β*-galactosidase genes were unevenly distributed on its four chromosomes (4, 6, 11, 12; Fig 3). In *A*. *fumigatus*, *β*-galactosidase genes were randomly distributed on three chromosomes (2 on chr1, 1 each on chr3 & 6), out of 8 chromosomes. Two genes of *β*-galactosidase identified in *F*. *fujikuroi* were present on its chromosome number 9 & 10, out of 12 chromosomes. For *A*. *oryzae*, 4 *β*-gal genes were distributed on three of its chromosomes (1 each on chr2 & 4, 2 on chr5), out of 8 chromosomes.

### 3.4 Domain & motif analysis and gene structure of *β*-gal genes

**Fig 4:**
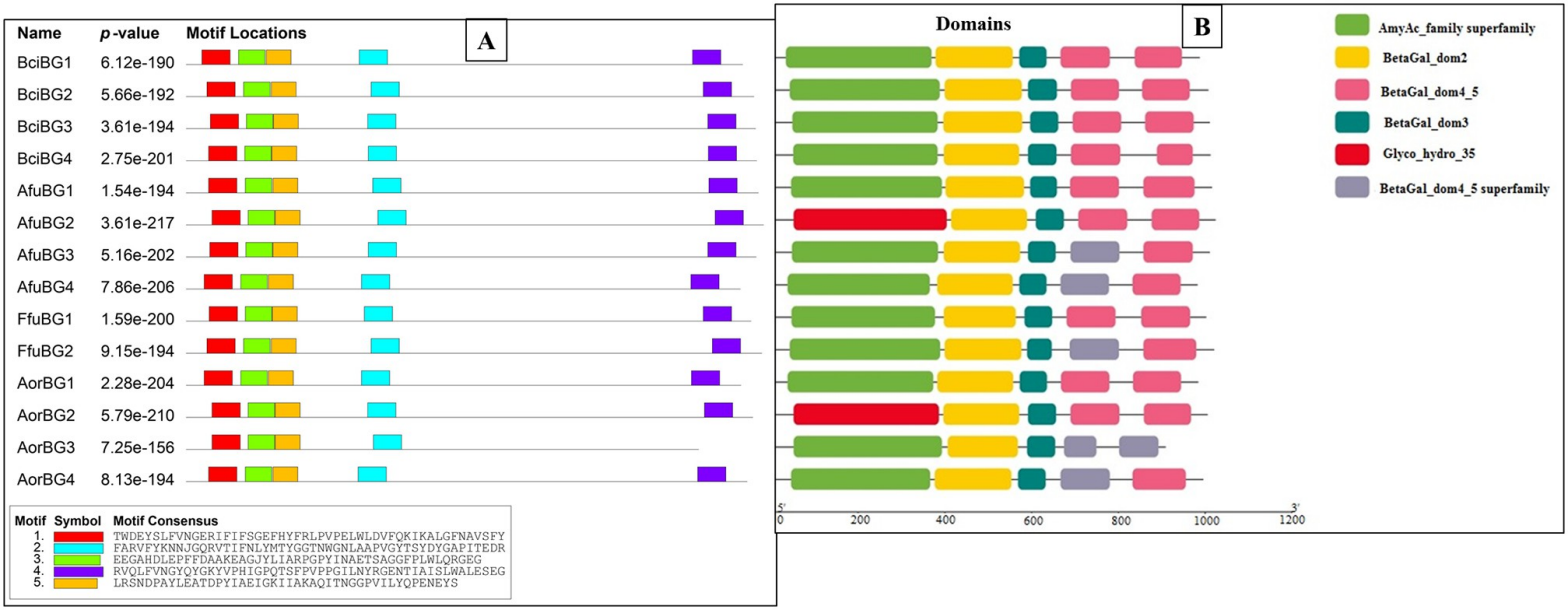
(A) Motif composition by MEME web server (5 motifs in all except AorBG3) and (B) Domain analysis using TBTOOLS (presence of 5 domains in all) of *β*-gal genes.

A total of 5 motifs were found in all proteins at approximately the same locations except for AorBG3 which lacked one motif. Domain analysis showed the presence of Glyco-hydro-35 domain, BetaGal domain 2, domain 3 & domain4_5 in all proteins. AmyAc-superfamily domain includes Glyco-hydro-35 domain and most proteins are denoted by AmyAc-superfamily. Presence of Glyco-hydro-35 domain is also validated by Pfam webserver. Motif locations along with consensus sequences and domains are shown in Fig 4.

**Fig 5:**
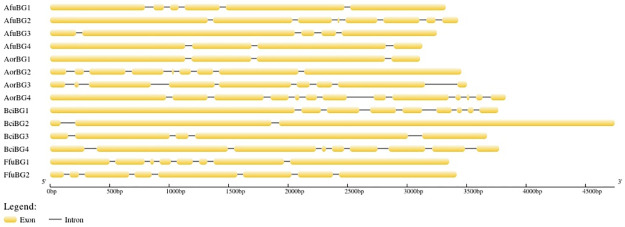
Gene structure prediction of *β*-gal genes. Exons are represented with yellow pipelines and black solid lines show the representation of introns.

Gene structure prediction showed the presence of exonic and intronic regions but no un-translated regions (UTR) at 3’ and 5’ ends (Fig 5). AorBG4 contained the most introns & exons (12 introns and 13 exons) and BciBG2 had the least introns and exons (2 introns and 3 exons) among all. Least number of introns are favorable as they require less energy expedition during splicing and reduces the chance of splicing errors.

### 3.5 Synteny analysis of *β*-gal genes

**Fig 6:**
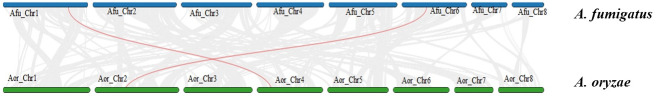
Synteny analysis of *β*-gal genes of *A*. *fumigatus* and *A*. *oryzae*. Red lines indicate the presence of a synteny/evolutionary relationship for *β*-gal genes and gray lines indicate synteny of all other genes of their genome. No synteny was observed in all other pairs of organisms with respect to *β*-gal.

TBTools used to perform synteny analysis of *β*-gal genes of *B*. *cinerea*, *A*. *fumigatus*, *A*. *oryzae* and *F*. *fujikuroi* with each other, revealed the presence of homologous syntenic blocks in the genomes of *A*. *fumigatus* and *A*. *oryzae* only (Fig 6). Our analysis identified 2 homologous gene pairs in *A*. *fumigatus* and *A*. *oryzae* genomes while no homologous gene pairs, for *β*-gal genes, were identified in all other pairs of organisms. The evolutionary homology relationship of *β*-gal genes was indicated between *A*. *fumigatus* and *A*. *oryzae*.

### 3.6 Physicochemical properties and sub-cellular localization

Physicochemical properties of *β*-gal of *B*. *cinerea*, *A*. *fumigatus*, *A*. *oryzae & F*. *fujikuroi* were evaluated. Complete details of physicochemical properties of aforementioned fungal proteins are mentioned in Table 1. The results indicated the molecular weights of *β*-gal of different fungal species varied from 100788.42 of AorBG3 to 113449.54 of FfuBG2 and the isoelectric points (pI) reside in the acidic range except that of FfuBG1(basic). All proteins were found to be stable as the instability index was less than 40. A high aliphatic index (>40%) demonstrated the thermal stability of proteins. By comparing each species’ proteins within species, BciBG3 and AfuBG2 found to be most stable but interestingly, FfuBG1 has high stability but less thermal stability than FfuBG2 and AorBG3 has high thermal stability but more instability index than AorBG2. And among all proteins, AfuBG2 was found to be most stable. Negative values of GRAVY (Grand average of hydropathicity) index showed the polarity of these proteins with BciBG1 having least polarity and FfuBG2 having highest polarity. The different values indicate the diversity and uniqueness of different *β*-gal proteins.

**Table 1:** Physicochemical properties of *β*-gal proteins of understudied fungi indicated their thermal stability, non-polarity and acidic pH.

Proteins	Molecular weight	pI	Positive residues	Negative residues	Instability index	Aliphatic index	GRAVY index
BciBG1	108124.62	5.04	62	84	33.13	78.34	-0.177
BciBG2	110543.71	4.59	57	106	39.55	75.36	-0.242
BciBG3	111784.29	5.6	72	94	28.63	79.65	-0.262
BciBG4	109825.61	5.42	72	88	28.14	77.59	-0.201
AfuBG1	111680.86	5.47	75	97	32.85	75.07	-0.282
AfuBG2	112421.62	6.14	89	97	24.32	79.41	-0.277
AfuBG3	111734.36	6.09	85	96	34.47	77.07	-0.329
AfuBG4	108034.53	6.03	79	89	27.39	72.8	-0.345
FfuBG1	110420.64	9.05	112	97	24.62	76.67	-0.378
FfuBG2	113449.54	6.45	100	107	27.66	78.49	-0.394
AorBG1	108652.87	5	73	105	34.65	75.52	-0.318
AorBG2	109869.81	5.33	83	108	26.93	77.84	-0.306
AorBG3	100788.42	5.1	59	95	35.81	79.38	-0.323
AorBG4	109370.74	4.93	69	111	28.92	78.77	-0.303

**Fig 7:**
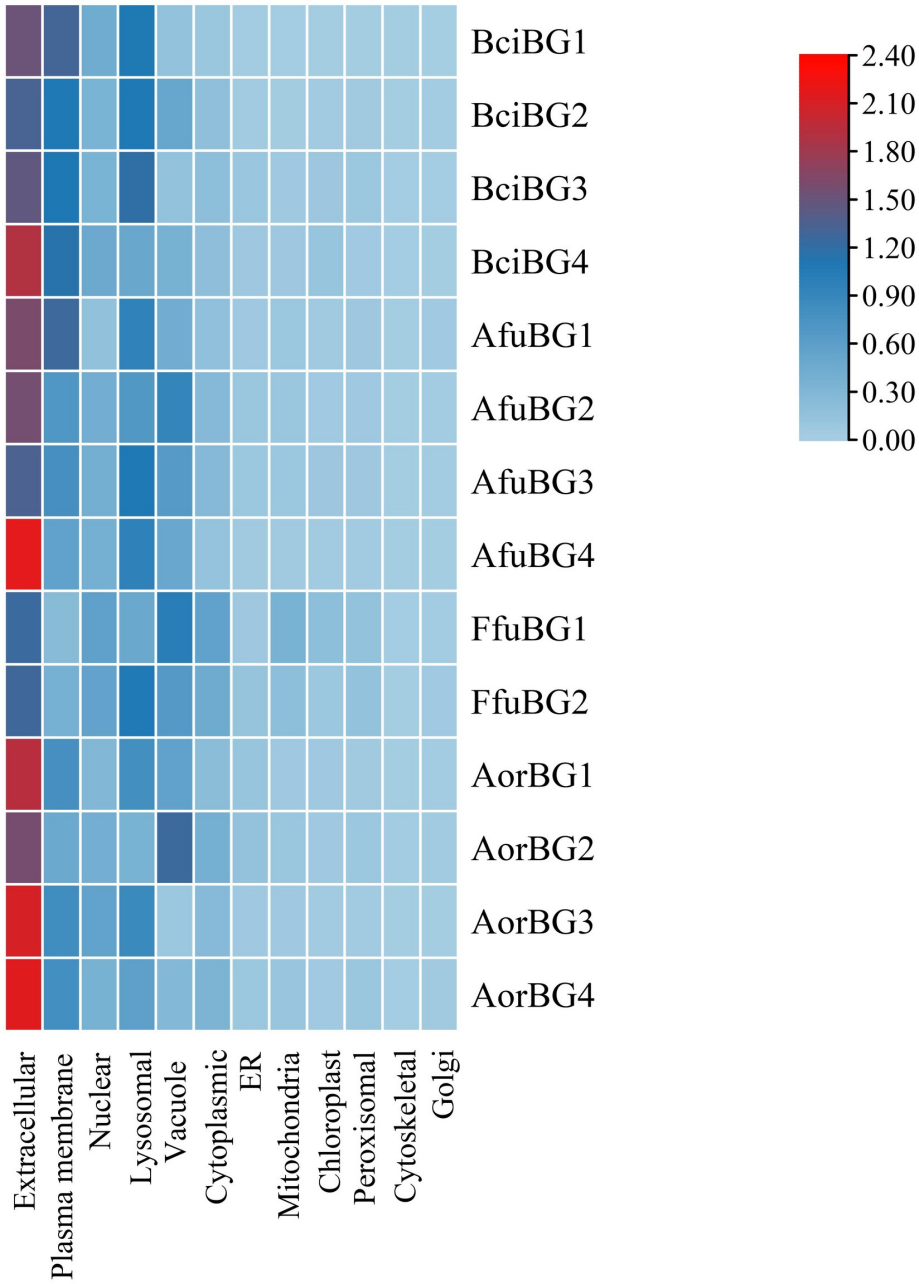
Sub-cellular localization of all *β*-gal proteins (generated by WoLF PSORT) by heat map illustration. All proteins were found to localized extensively in the extracellular region.

Sub-cellular localization was determined to predict the cellular location of all *β*-gal proteins of the four fungal species. The results suggested that the *β*-gal proteins are expressed (Fig 7) and extensively localized in extracellular region. These results suggested that extracellular localization will allow their easy *in-vitro* extraction, removing the barriers of utilizing sophisticated methods for otherwise intracellularly localized proteins.

### 3.7 Gene ontology of *β*-gal genes

**Fig 8:**
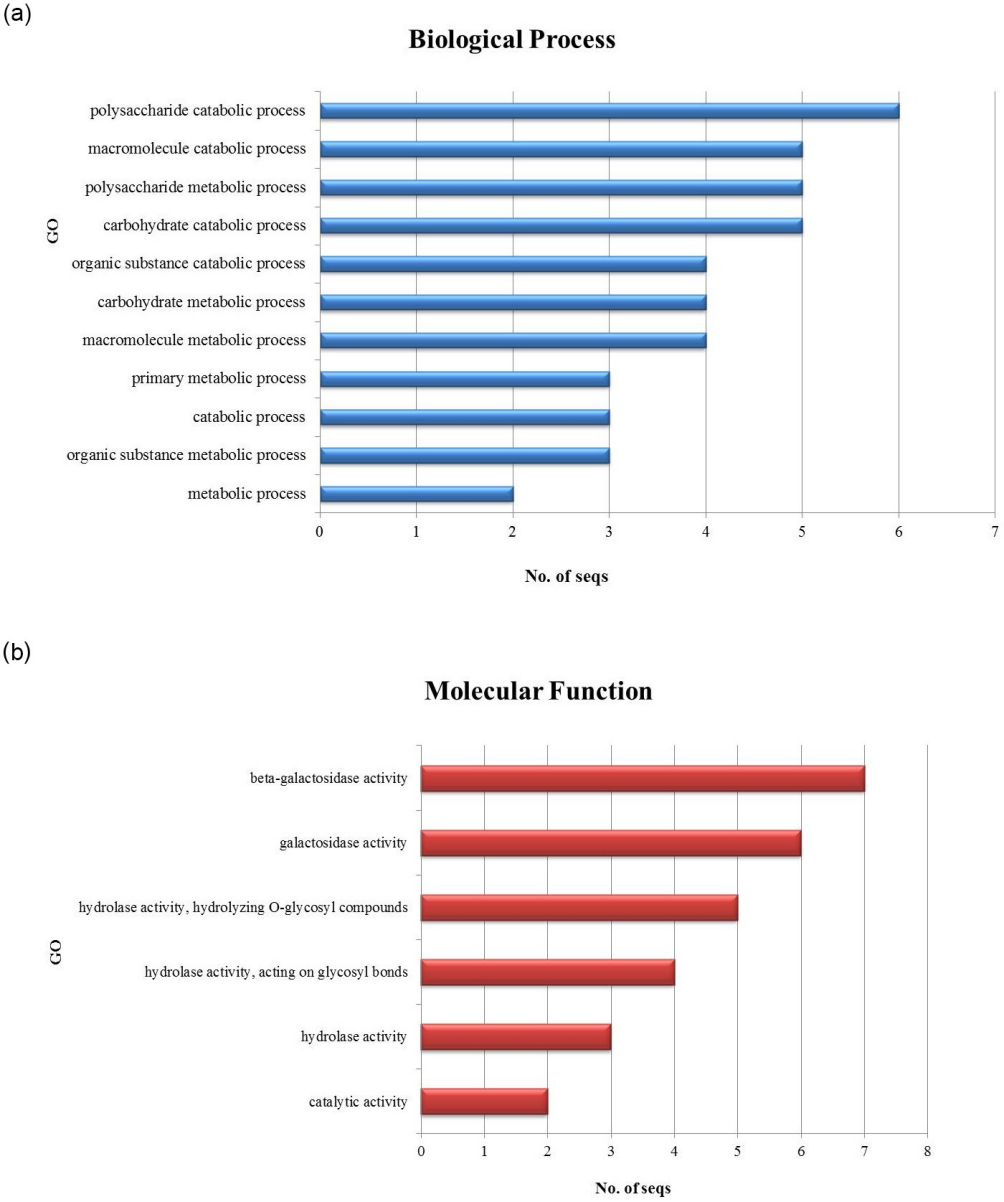
Gene ontology of *β*-gal genes by Blast2GO. (a) Biological processes are shown by blue bars and (b) molecular functions are shown by red bars.

Gene ontology, performed by Blast2GO tool, indicated that the biological processes of these genes have a role in the polysaccharide catabolic process (GO:0000272) and the molecular functions indicated to have their possible role in beta-galactosidase activity (GO:0004565). Data is given in S3 File and Ontology graphs are shown in Fig 8.

### 3.8 Signal peptide prediction of *β*-gal proteins

Signal peptides were found in all proteins except for AorBG3. Signal peptides, present at the start of protein sequences, direct the protein transfer to a specific sub-cellular location but in structure predictions, they must be removed to obtain pure enzyme structure. The cleavage sites, Sec/SPI, and signal peptide lengths of understudied enzymes obtained by SignalP are given in S2 File.

### 3.9 Structure prediction of identified *β*-gal proteins

**Fig 9:**
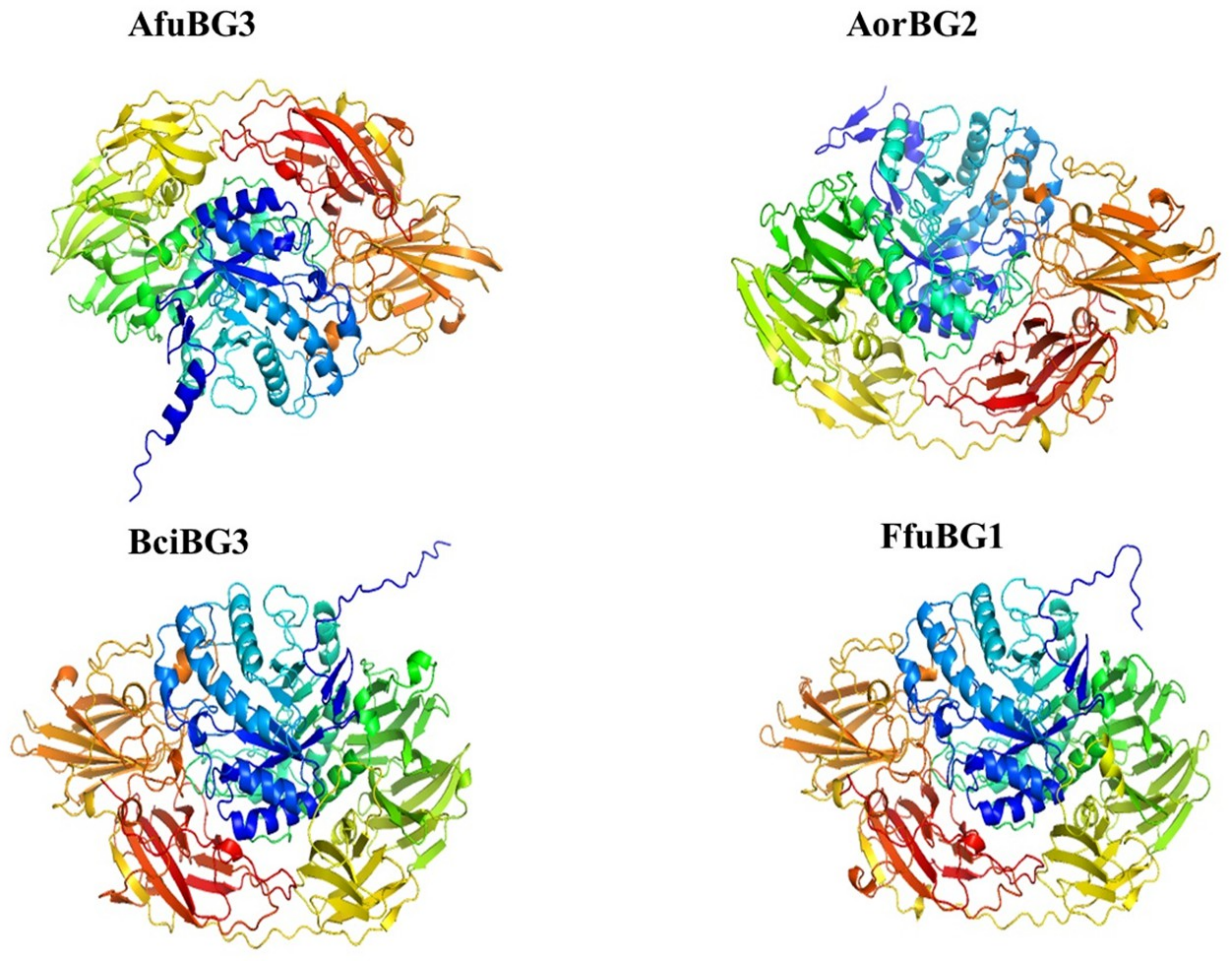
Selected protein models of *Aspergillus fumigatus* (*AfuBG3*), *Aspergillus oryzae* (*AorBG2*), *Botrytis cinerea* (*BciBG3*) *& Fusarium fujikuroi* (*FfuBG1*). Coils represent *α*-helices, arrow-headed broad lines represent *β*-strands and thread-like lines represent loops and turns.

**Fig 10:**
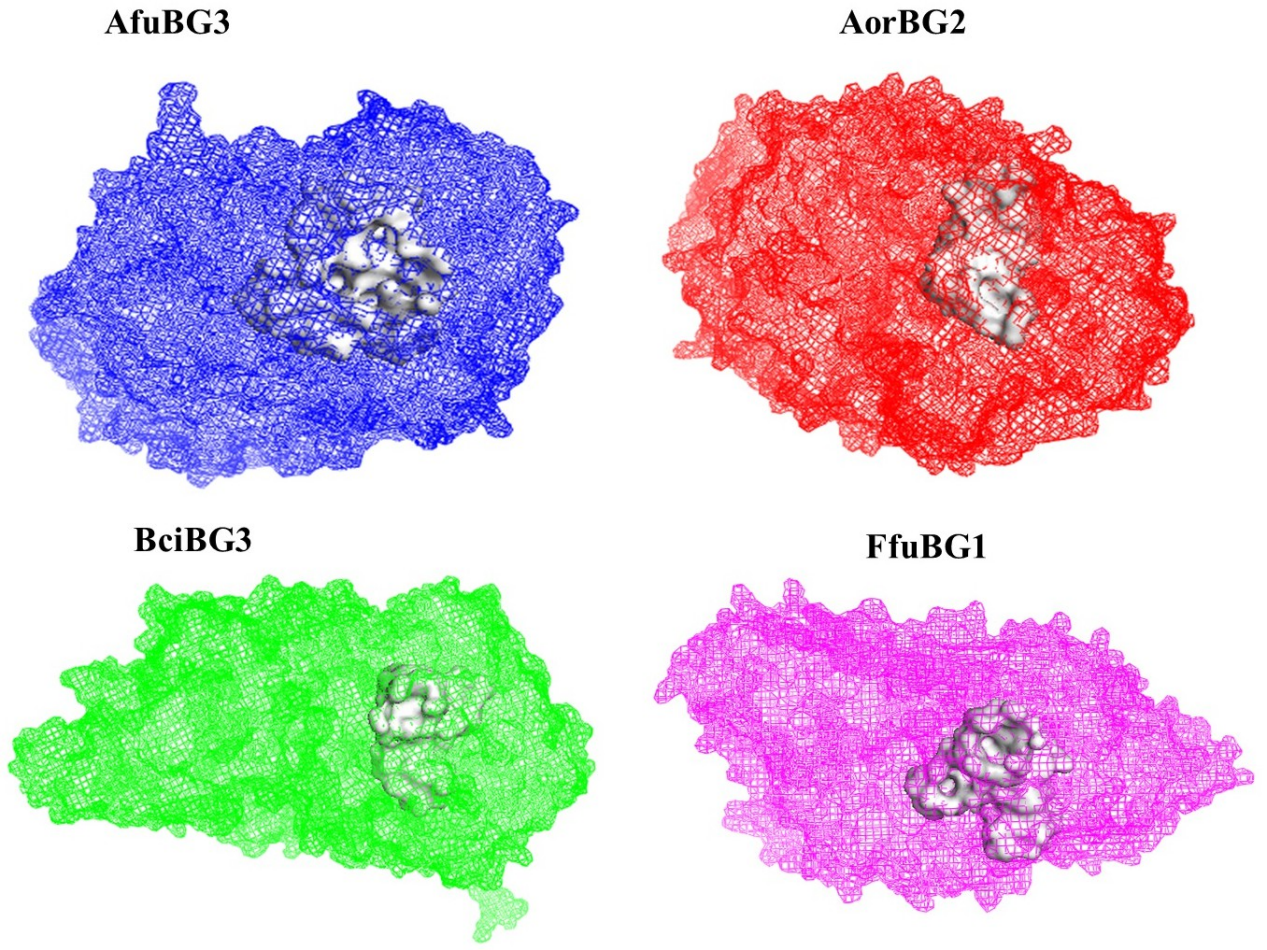
Predicted binding pockets of refined protein models are shown in white within all models. Glycine and leucine are the predominant residues. Blue model is of *Aspergillus fumigatus*, red model is of *Aspergillus oryzae*, green model is of *Botrytis cinerea* and magenta model is of *Fusarium fujikuroi*.

Structure prediction, performed by Robetta, end up with 4 best protein models; one for each species. These protein structures were then refined by the 3Drefine webserver. Refined protein modeling files are given in S4 File. Ramachandran plots used in model selection are given in S5 File. Attributes of selected protein structures are given in Table 2 and protein structures are shown in Fig 9. Binding sites predicted by DoGSiteScorer webserver for the refined protein models are shown in Fig 10.

**Table 2:** Ramachandran plots, ERRAT and Q-mean values of selected models of proteins.

Protein type	Tool	Method	Q-mean	ERRAT	Favorable regions (%)	Additional allowed regions (%)	Generously allowed regions (%)	Disallowed regions (%)
AfuBG3	Robetta	RoseTTA fold	0.77	86.4476	86.6	12.2	0.6	0.6
AorBG2	Robetta	RoseTTA fold	0.81	90.7598	86.8	11.9	0.5	0.8
**FfuBG1**	**Robetta**	**RoseTTA fold**	**0.78**	**90.7407**	**86.1**	**12.6**	**0.8**	**0.5**
BciBG3	Robetta	RoseTTA fold	0.75	88.2474	87	11.7	0.7	0.6

*Fusarium fujikuroi*’s modeled *β*-gal protein is predicted to be most stable than that of the others, nevertheless, all modeled *β*-gal proteins have the potential to exist stably.

Abundant active residues in protein models were found to be leucine and glycine. Small and shallow binding pockets were predicted consisting of 20, 22, 25 and 27 amino acids in *Botrytis cinerea*, *Aspergillus oryzae*, *Fusarium fujikuroi* and *Aspergillus fumigatus* respectively, data given in S6 File.

**Fig 11:**
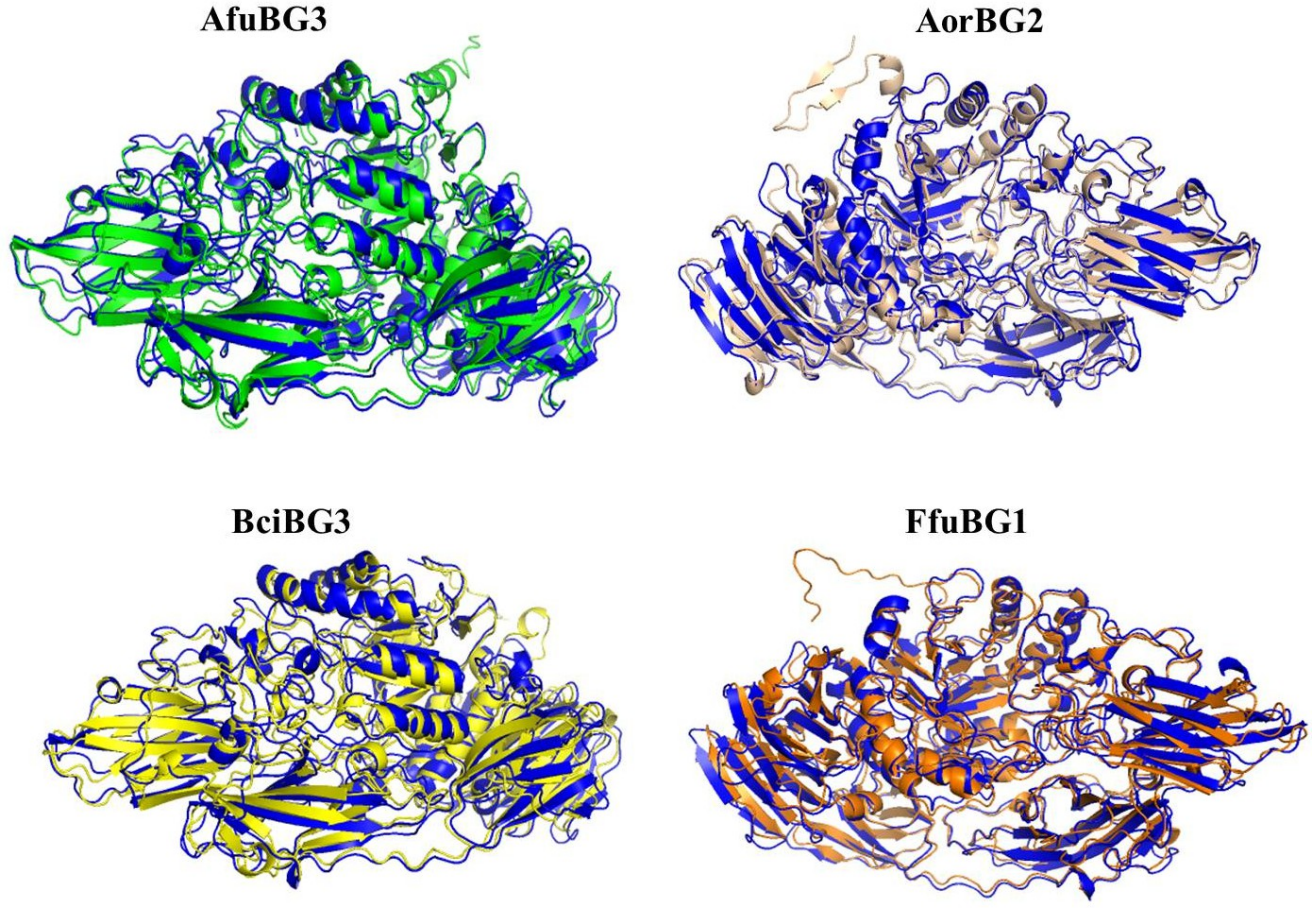
Superimposition of selected protein models with *Aspergillus oryzae* β-gal structure from RCSB PDB (4IUG) in PyMOL. Blue structure is of 4IUG, superimposed with green (*A*. *fumigatus*), wheat (*A*. *oryzae*), yellow (*B*. *cinerea*) and orange (*F*. *fujikuroi*) colored structures.

Selected protein models were superimposed with *Aspergillus oryzae β*-gal structure (4IUG) from RCSB PDB (https://www.rcsb.org/) in PyMOL as shown in Fig 11. RMSD values for selected *Aspergillus oryzae*, *Aspergillus fumigatus*, *Botrytis cinerea* and *Fusarium fujikuroi β*-gal proteins with 4IUG were found to be 1.482Å, 1.648Å, 1.660Å and 1.510Å respectively. Lower RMSD values indicated the significant superimposition with high similarity between these structure groups.

### 3.10 Differential expression analysis of *β*-gal genes of *A*. *fumigatus* on GPA and MM media

**Fig 12:**
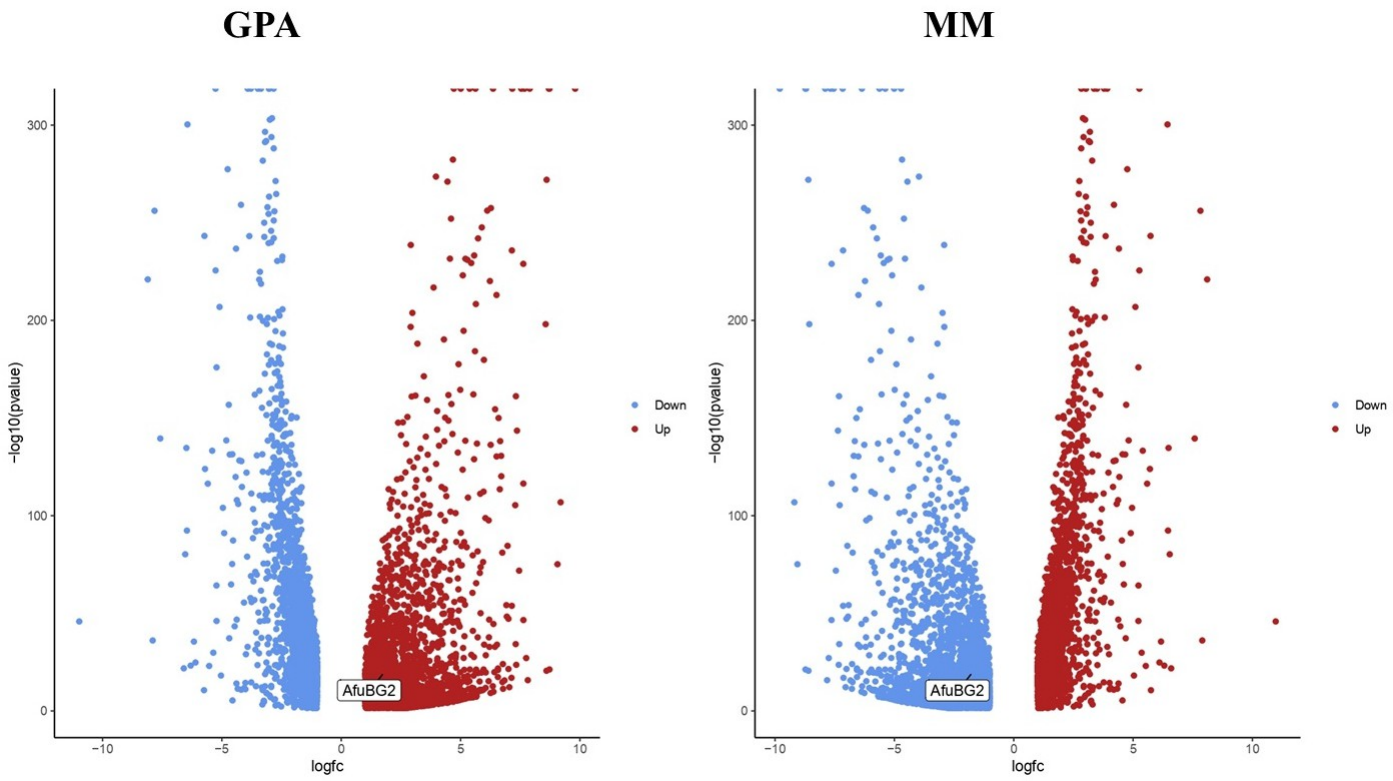
Differential gene expression of *A*. *fumigatus* β-gal gene (AfuBG2) on GPA and MM media. AfuBG2 (logfc = 1.76; p-value = 6.42e^-20^; p-adj = 2.45e^-19^) is up-regulated on the GPA medium (left side) and AfuBG2 (logfc = -1.76; p-value = 6.42e^-20^; p-adj = 2.45e^-19^) is down-regulated on the MM medium (right side) for each other.

RNA-sequencing analysis of *A*. *fumigatus* under GPA and MM media was performed using the GALAXY webserver to have insights about the possible role of *β*-gal in the germination of *A*. *fumigatus*. DESeq2 tool was used to analyze up-regulated and down-regulated *β*-gal genes in GPA and MM media, given in S7 File. Volcano plots showed the differential gene expression as shown in Fig 12. Among the four *A*. *fumigatus β*-gal genes, AfuBG2 was found to be up-regulated on GPA medium (logfc = 1.76; p-value = 6.42e^-20^; p-adj = 2.45e^-19^) and down-regulated on MM medium (logfc = -1.76; p-value = 6.42e^-20^; p-adj = 2.45e^-19^) for each other while other *β*-gal genes (AfuBG1, AfuBG3 and AfuBG4) had not any significant differential expression in the current scenario. Growth in GPA and MM media accounted for the difference in the time of germination of germinated conidial samples of *A*. *fumigatus*. Growth of germ tube was inhibited in GPA medium after 30h incubation (fungus was alive) in most of the conidia while it continued to grow in MM medium. This reduced growth was correlated with the starvation of fungus on GPA medium followed by the expression of genes involved in the survival of *A*. *fumigatus*. So, *β*-gal gene expression was up-regulated in GPA medium as it is a galactoside-cleaving enzyme for energy production and the fungus shifted to autolysis and became dormant for its survival. The germ tube inhibition in GPA medium with the up-regulation of AfuBG2 gene concludes the inverse relation between AfuBG2 and germ tube development. So, a competent media (nutritious conditions) is essential for *A*. *fumigatus* to grow properly (developed germ tube) as evidenced by the resumption of its normal growth when it is exposed to MM medium after its inhibition mode on GPA medium.

Furthermore, change in the growth medium allowed the differential expression of the *β*-gal gene suggesting that the appropriate medium is required for optimum production of the *β*-galactosidase enzyme. And in this case, the GPA medium was more appropriate than the MM medium for *β*-gal production, owing to the survival needs of the fungus.

## 4. Discussion

Enzymes have a crucial role in everyday life processes and industries. The *β*-galactosidase enzyme is one of the key players in the dairy industry along with the potential applications in medical and analytical sectors [1]. For its *in-vitro* isolation & having genetic and proteomic insights, *in-silico* studies aid by reducing time and resources. Currently, besides this study, no such comprehensive *in-silico* study regarding fungal *β*-galactosidases has been reported. Out of 14 *β*-gal genes (GH-35) identified in this study, genes of *B*. *cinerea* (4) and *A*. *oryzae* (4) were reported here for the first time. *β*-gal genes have already been reported in fungi; 5 genes in *Aspergillus niger*, 5 genes in *P*. *chrysogenum*, gene(s) in *Aspergillus sp*., *Penicillium sp*., *Trichoderma reesei*, *Talaromyces cellulolyticus* and yeasts [31–37]. Phylogenetic analysis, established among the *β*-gal of understudied strains and *Neurospora crassa*, *Aspergillus fischeri*, *Penicillium digitatum* and *Fusarium oxysporum β*-galactosidases with *Rhizoctonia solani β*-gal as outgroup, revealed that *β*-gal genes of *A*. *fumigatus* and *A*. *oryzae* were sharing the same clades and *F*. *fujikuroi* genes were sharing clades with *F*. *oxysporum* and *N*. *crassa*. *B*. *cinerea* genes were closely related to *A*. *fumigatus* and *A*. *oryzae* genes. Besides, having the evolutionary relationships among same fungal genera, evolutionary patterns were seen among different fungal genera too. Likewise, synteny was found between *A*. *fumigatus* and *A*. *oryzae* genes only due to the evolutionary closeness of these two species. The *β*-gal genes were randomly distributed on different chromosomes but *B*. *cinerea* and *A*. *oryzae* had a common chromosome (4) and *B*. *cinerea* and *A*. *fumigatus* had a common chromosome (6) for *β*-gal genes and is evidenced by their evolutionary insights *via* phylogenetic analysis. And this might be due to the evolutionary mechanism of horizontal gene transfer among these species that leads to the presence of *β*-gal genes on the same chromosomes [38]. Motif & domain analysis highlighted 5 motifs & domains each at approximately the same locations except AorBG3. Domains found in understudied fungi are similar to the ones reported in other GH-35 *β*-galactosidases. For instance, *Aspergillus niger* (AAC60538.1), *Penicillium digitatum* (XP_014530847.1), *Fusarium oxysporum* (EXK29145.1), *Aspergillus piperis* (RAH52028.1) and *Rhizoctonia solani* (EUC61741.1) *β*-galactosidases share same domains.

Proteins identified in this study were predicted to be residing in the extracellular area, like most of the fungal *β*-galactosidases. *Aspergillus niger*, *Hypocrea jecorina*, and *Guehomyces pullulans* have extracellular *β*-gal [31, 34, 39] while *P*. *chrysogenum* has both extracellular and intracellular *β*-gal [32]. Extracellular compartmentalization has the competitive advantage in the *in-vitro* isolation of *β*-gal as intracellular enzymes require various sophisticated techniques for their isolation, leading to the prolonged duration and effort in the process. The isoelectric points predicted the fungal *β*-galactosidases to be acidic (except one) in the current study. Similarly, *A*. *niger* and yeast, *Guehomyces pullulans* have acidic *β*-galactosidases [31, 34]. Acidic nature of identified fungal *β*-gal mimics the working pH of human *β*-gal, leading to its smooth application in the human body. The *β*-galactosidases were predicted to be thermally stable due to their lower instability index and higher aliphatic index. The sum of hydropathy values of all amino acids, divided by the number of residues in the protein sequence constitutes its GRAVY value. Hydrophilicity and hydrophobicity of a protein is given by negative and positive GRAVY values respectively [40]. *β*-galactosidases were predicted to be polar in nature due to their hydrophilic nature (negative GRAVY). Polar enzymes have the ability to better interact with water and are thus soluble, validating the freely presence of *β*-gal in extracellular region. The GRAVY values of *β*-galactosidases from *Bacillus* sp. also fall in the hydrophilic range (-0.517 to -0.452), indicating their polar nature [41].

Exonic/intronic ratio in *β*-gal genes was found to be variable with BciBG2 having the least introns and AorBG4 having the most introns; it predicted the comparative rapid production of BciBG2 and delayed production of AorBG4 enzymes due to less hindrance (in terms of energy and errors) in the splicing process and consequently in translation of BciBG2. The high intronic burden has a direct link with the evolutionary conserveness of a gene, indicating the fact that the necessity of the gene overcomes the increased intronic hindrance in gene expression [42]. And due to high intronic ratio, unique splice variants can be produced according to the needs of the organism. On the other hand, due to the presence of highly distributed and non-linked *β*-gal genes in all understudied species (chromosomal distribution), it is possible that *β*-gal genes might get redundant over the course of time [43]. But because of the evolutionary importance of *β*-gal, this actually might not happen. This concludes the evolutionary importance of *β*-gal. It has been reported that *A*. *niger* has 5, 5, 8, 6 & 11 introns in 5 of its *β*-gal genes [31], so it might be possible that *Botrytis cinerea β*-gal has the potential of rapid production than that of *A*. *niger β*-gal too.

Here, we have modeled novel *β*-gal structures of *Aspergillus fumigatus*, *Aspergillus oryzae*, *Botrytis cinerea & Fusarium fujikuroi* from Robetta webserver, by selecting the best models on the basis of ERRAT, Q-MEAN and Ramachandran plot values. ERRAT analysis identified the quality factor of modeled proteins by taking into account the interaction patterns (non-bonded) among different types of atoms [44]. Energy stabilization was predicted *via* Q-MEAN analysis [45] and further selection of proteins was done based on their residual locations in favorable or disallowed regions, i.e., Ramachandran plot values [46]. The prediction of higher ERRAT (86.4476, 90.7598, 88.2474 and 90.7407) and Q-MEAN (0.77, 0.81, 0.75 and 0.78) values along with the greater percentage of most favorable region residues (86.6, 86.8, 87 and 86.1%) and lower percentage of disallowed region residues (0.6, 0.8, 0.6 and 0.5%) for modeled *β*-gal structures of *A*. *fumigatus*, *A*. *oryzae*, *B*. *cinerea & F*. *fujikuroi* respectively, proposed them as stable structures. The predicted Q-MEAN values are greater than the reported Q-MEAN values of *Aspergillus niger*’s *β*-galactosidases, i.e., 0.725, 0.657, 0.692, and 0.722 [31]. And the Ramachandran plot values of *A*. *oryzae β*-gal [47] are somewhat similar to the predicted values of this study (most favorable region residues-87.8%, additional allowed region residues-11.5%, generously allowed region residues-0.4% and disallowed region residues-0.3%).

Regarding crystal structures of fungal *β*-galactosidases, structures of *Aspergillus niger* [48], *Aspergillus oryzae* [47], *Kluyveromyces lactis* [35], *Penicillium sp*. [36] and *Trichoderma reesei* [37] have been reported in protein data bank. By comparing the selected *β*-gal sequences with the fungal *β*-gal structures in PDB, *A*. *fumigatus β*-gal represents 53–54% homology with *A*. *oryzae* and *A*. *niger β*-gal, *A*. *oryzae β*-gal shows 99.9% homology with reported *A*. *oryzae β*-gal and 75% homology with *A*. *niger* one, *β*-gal of *B*. *cinerea* exposes approximately 50% homology with *β*-gal of *A*. *oryzae*, *A*. *niger* and *Penicillium* sp. and finally the *F*. *fujikuroi β*-gal represents 62% homology with *Trichoderma reesei β*-gal, followed by 54–56% homology with *A*. *oryzae*, *A*. *niger* and *Penicillium* sp. *β*-gal. A reasonable similarity of *A*. *oryzae β*-gal (4IUG) with the selected *β*-galactosidases was observed. The reported *Aspergillus oryzae β*-gal is different from our modeled *A*.*oryzae β*-gal, owing to its different gene origin. Binding pockets of our refined models were predicted to be shallow and small, having predominant leucine and glycine residues. Glu-537 is known to be the main attachment center for lactose in *E*. *coli β*-galactosidase [49]. Glu-200 and Glu-298 in *Aspergillus oryzae* [47], *Aspergillus niger* [48] and *Trichoderma reesei* [37], Glu-200 and Glu-299 in *Penicillium sp*.[36] and Glu-482 and Glu-551 in *Kluyveromyces lactis* [35] constitute the catalytic residues. So, it might be possible that Glu-559 predicted in our modeled *Aspergillus oryzae* can be the main attachment site for the ligand and Glu-81 and Glu-838 can also have the same fate as predicted in *Fusarium fujikuroi*. In order to have the comparative structural insights into the refined protein models, they each were superimposed with the reported protein structure of *Aspergillus oryzae* from RCSB PDB. The resultant lower RMSD values of all superimpositions (less than 4) indicated the significant superimposition with high similarity between them. This highlights the structural liability of selected protein models [50] and also verifies the difference between selected *Aspergillus oryzae β*-gal protein and *Aspergillus oryzae β*-gal protein from protein data bank.

The role of identified fungal *β*-galactosidase genes is predicted to be found in polysaccharide catabolic process and beta-galactosidase activity. This is in parallel to the known *β*-galactosidase systematics, further validating the nature of identified genes. RNA-sequencing analysis has been performed on *β*-galactosidase genes of *Aspergillus fumigatus* by comparing expression profiles of *A*. *fumigatus* on GPA and MM media [30]. This investigation also revealed a novel inverse relationship of *β*-gal genes in germ tube development and suggested that a less nutritious medium (GPA) is favorable for enhanced production of *β*-gal, owing to the survival needs of *A*. *fumigatus*. Conidia after being exposed to certain nutritious conditions are set free from dormancy and pass onto the isotropic growth stage (swollen state by absorbing water), followed by the polarized growth pattern (unidirectional growth) that leads to the germ tube formation. Germ tubes differentiate and grow into hyphae and are known to play a role in the pathogenesis of fungi [25, 27]. Invasive aspergillosis is an infectious condition in immune-compromised patients, mainly infecting the lungs but can be spread to other parts of the body, that is chiefly caused by *A*. *fumigatus* [26]. A study on the human cell line of lung epithelium A549, indicated the enhanced release of inflammatory mediator genes by exposure to *A*. *fumigatus* germ tube growth rather than conidia ingestion [27], indicating the link between germ tube and the infection.

So, it might be possible to have a less virulent strain of *A*. *fumigatus* by providing the conditions that will favor the *β*-gal production (starving conditions) and thus leading to less severe condition of the disease, due to delaying or inhibition of the germ tube development. Another interesting fact; due to the starving conditions and halting of metabolic pathways, galactosaminogalactan (GAG), having a crucial role in the pathogenesis of *A*. *fumigatus* [51], was not produced in a considerable amount [30]. The greater expression of *β*-gal might lead to less production of GAG and thus it concludes the less virulent state of Aspergillosis. Besides, GPA medium was found to be more active in the production of *β*-gal than MM medium, indicating the fact that a less nutritious medium is favorable for the *β*-gal production as it is an energy producing (glucose and galactose) enzyme so it is active in the survival mode of *A*. *fumigatus*. So, it seems to have an advantage of obtaining more *β*-gal by providing fewer nutrients.

To conclude, all the selected fungal species were found to be the potential candidates for the *β*-gal production with some species having edge over the others, in terms of exonic/intronic ratio and certain physicochemical properties. The *in-silico* characterizations of fungal β-galactosidases will help in the β-gal genetic and proteomic modifications in future endeavors and it also leaves a gap for the *in-vitro* validations in future research avenues. To have better insights into the role of *β*-gal in germ tube development and eventually in Aspergillosis, specific *in-vitro* studies, including the comparison of the wild type *A*. *fumigatus* and the *β*-gal knock-down *A*. *fumigatus* strain, is required.

## 5. Conclusion

*In-silico* identification and computational comparison of *β*-galactosidase (GH-35) in *Aspergillus fumigatus*, *Aspergillus oryzae*, *Botrytis cinerea & Fusarium fujikuroi* revealed 14 *β*-gal genes (four each in *B*. *cinerea*, *A*. *fumigatus*, and *A*. *oryzae* & two in *F*. *fujikuroi*). The predicted proteins were predicted to be thermally stable, acidic, non-polar, extra-cellularly localized, and involved in polysaccharide catabolic process. Furthermore, best protein models of *β*-gal have been structured based on the higher ERRAT and Q-MEAN values as well as the greater percentage of favorable region residues. Finally, the inverse role of *β*-gal in germ tube development of *A*. *fumigatus* is elucidated by RNA-sequencing analysis.

## Supporting information

S1 File Table containing identified *β*-gal genes in *A*. *fumigatus*, *A*. *oryzae*, *B*. *cinerea & F*. *fujikuroi* along with different attributes. (DOCX)

S2 File Table containing cleavage sites and Sec/SPI values of proteins given by SignalP. (DOCX)

S3 File It contains gene ontology data. (ZIP)

S4 File It contains pdb files of selected refined models of *β*-gal of *A*. *fumigatus*, *A*. *oryzae*, *B*. *cinerea & F*. *fujikuroi*. (ZIP)

S5 File It contains Ramachandran plots, used for the selection of *β*-gal protein structures. (ZIP)

S6 File It contains binding pocket data of protein models. (ZIP)

S7 File It contains DESeq2 resultant data of RNA-seq analysis. (ZIP)

S1 Graphical abstract (TIF)
