# Supplementary material for: Genome-wide identification and comparative in-silico characterization of β-galactosidase (GH-35) in ascomycetes and its role in germ tube development of Aspergillus fumigatus via RNA-seq analysis
Source: PLoS One. 2023 Jun 22;18(6):e0286428. doi: 10.1371/journal.pone.0286428 (PMC10287015; doi:10.1371/journal.pone.0286428)
Supplement: S1 File — (DOCX) [file pone.0286428.s001.docx]

Table: Identified *β*-gal genes in *A. fumigatus, A. oryzae, B. cinerea* & *F. fujikuroi* along with different attributes.

| **Names of genes** | **Protein ID** | **Gene ID** | **Chromos-**  **ome (Chr)**  **ID** | **Chr No.** | **Start point** | **End point** | **Gene length (nt)** | **Amino acid length** |
| --- | --- | --- | --- | --- | --- | --- | --- | --- |
| AfuBG1 | XP_753037.1 | 3510063 | NC_007194.1 | 1 | 4545429 | 4548752 | 3324 | 1015 |
| AfuBG2 | XP_752787.1 | 3509797 | NC_007194.1 | 1 | 3783553 | 3786982 | 3430 | 1024 |
| AfuBG3 | XP_748360.1 | 3505787 | NC_007196.1 | 3 | 78222 | 81471 | 3250 | 1011 |
| AfuBG4 | XP_750558.1 | 3508765 | NC_007199.1 | 6 | 1446088 | 1449216 | 3129 | 983 |
| AorBG1 | XP_001819282.1 | 5991265 | NC_036436.1 | 2 | 2200157 | 2203265 | 3109 | 984 |
| AorBG2 | XP_001727461.1 | 5987935 | NC_036438.1 | 4 | 1114648 | 1118104 | 3457 | 1005 |
| AorBG3 | XP_023092277.1 | 5995622 | NC_036439.1 | 5 | 331267 | 334769 | 3503 | 909 |
| AorBG4 | XP_023092437.1 | 5996166 | NC_036439.1 | 5 | 3087224 | 3091052 | 3829 | 995 |
| BciBG1 | XP_024548479.1 | 5438362 | NC_037313.1 | 4 | 2069547 | 2073312 | 3766 | 987 |
| BciBG2 | XP_024549322.1 | 5439872 | NC_037315.1 | 6 | 1531798 | 1536542 | 4745 | 1007 |
| BciBG3 | XP_024551677.1 | 5437289 | NC_037320.1 | 11 | 722674 | 726345 | 3672 | 1010 |
| BciBG4 | XP_001557985.1 | 5438586 | NC_037321.1 | 12 | 106896 | 110669 | 3774 | 1012 |
| FfuBG1 | XP_023435913.1 | 35402810 | NC_036630.1 | 9 | 2560458 | 2563811 | 3354 | 1002 |
| FfuBG2 | XP_023436791.1 | 35404240 | NC_036631.1 | 10 | 1508037 | 1511453 | 3417 | 1021 |
