# Supplementary material for: Genome-wide identification and comparative in-silico characterization of β-galactosidase (GH-35) in ascomycetes and its role in germ tube development of Aspergillus fumigatus via RNA-seq analysis
Source: PLoS One. 2023 Jun 22;18(6):e0286428. doi: 10.1371/journal.pone.0286428 (PMC10287015; doi:10.1371/journal.pone.0286428)
Supplement: S2 File — (DOCX) [file pone.0286428.s002.docx]

Table: Cleavage sites and Sec/SPI values of proteins given by SignalP.

| **Proteins** | **Cleavage sites** | **Sec/SPI** | **Signal peptide length (amino acids)** |
| --- | --- | --- | --- |
| BciBG1 | Between 19&20 | 0.9945 | 19 |
| BciBG2 | Between 18&19 | 0.9875 | 18 |
| BciBG3 | Between 20&21 | 0.5161 | 20 |
| BciBG4 | Between 18&19 | 0.9606 | 18 |
| AfuBG1 | Between 21&22 | 0.9958 | 21 |
| AfuBG2 | Between 18&19 | 0.9962 | 18 |
| AfuBG3 | Between 19&20 | 0.6634 | 19 |
| AfuBG4 | Between 23&24 | 0.9982 | 23 |
| FfuBG1 | Between 17&18 | 0.9739 | 17 |
| FfuBG2 | Between 20&21 | 0.9962 | 20 |
| AorBG1 | Between 23&24 | 0.9992 | 23 |
| AorBG2 | Between 18&19 | 0.9936 | 18 |
| AorBG3 | None | 0.0012 | None |
| AorBG4 | Between 22&23 | 0.9476 | 22 |
