# Supplementary figures and images for: Genome-wide identification and comparative in-silico characterization of β-galactosidase (GH-35) in ascomycetes and its role in germ tube development of Aspergillus fumigatus via RNA-seq analysis
Source: PLoS One. 2023 Jun 22;18(6):e0286428. doi: 10.1371/journal.pone.0286428 (PMC10287015; doi:10.1371/journal.pone.0286428)

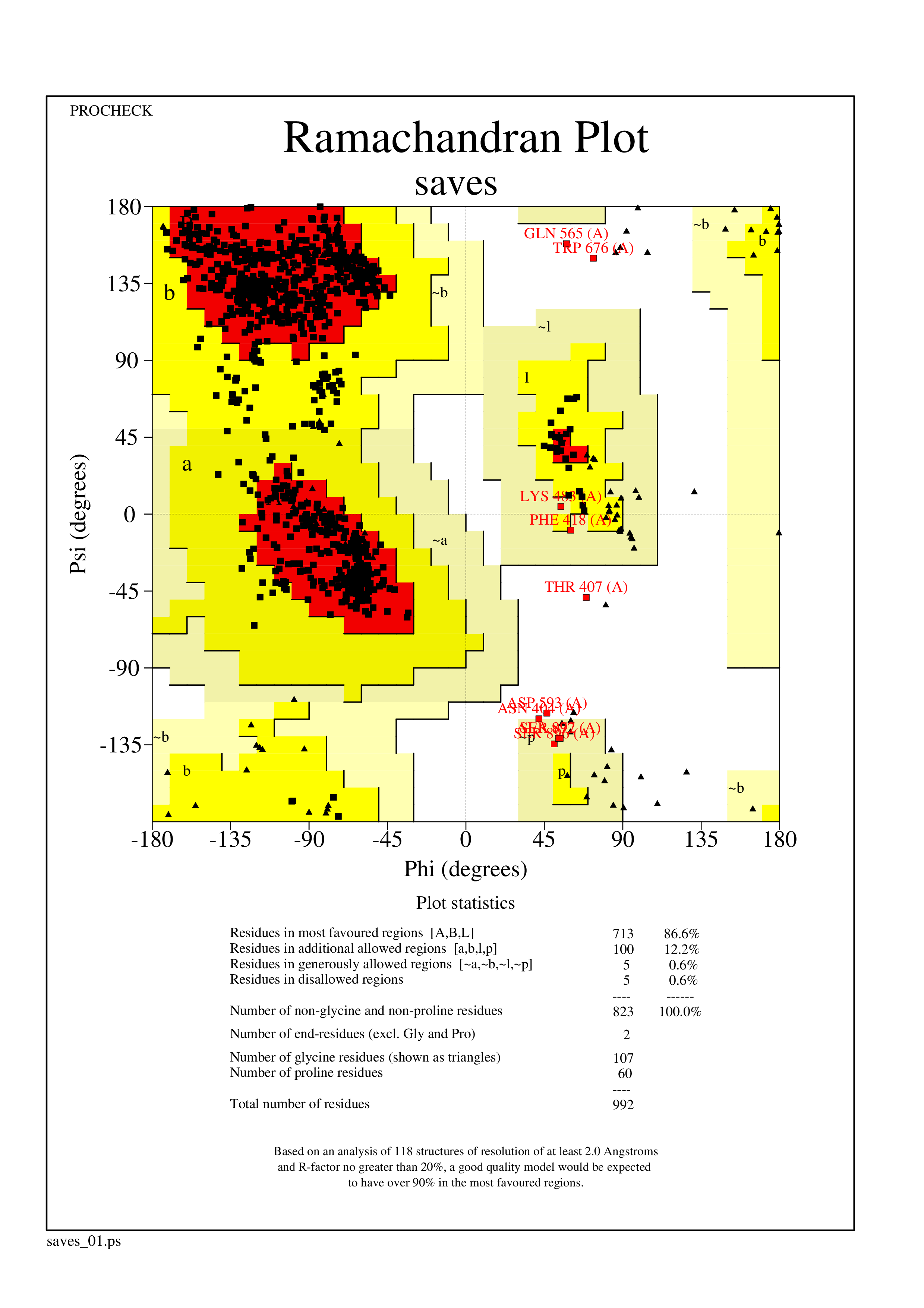

Supplement: S5 File — (ZIP) [file pone.0286428.s005.zip › AfuBG3-Ramachandran.png]

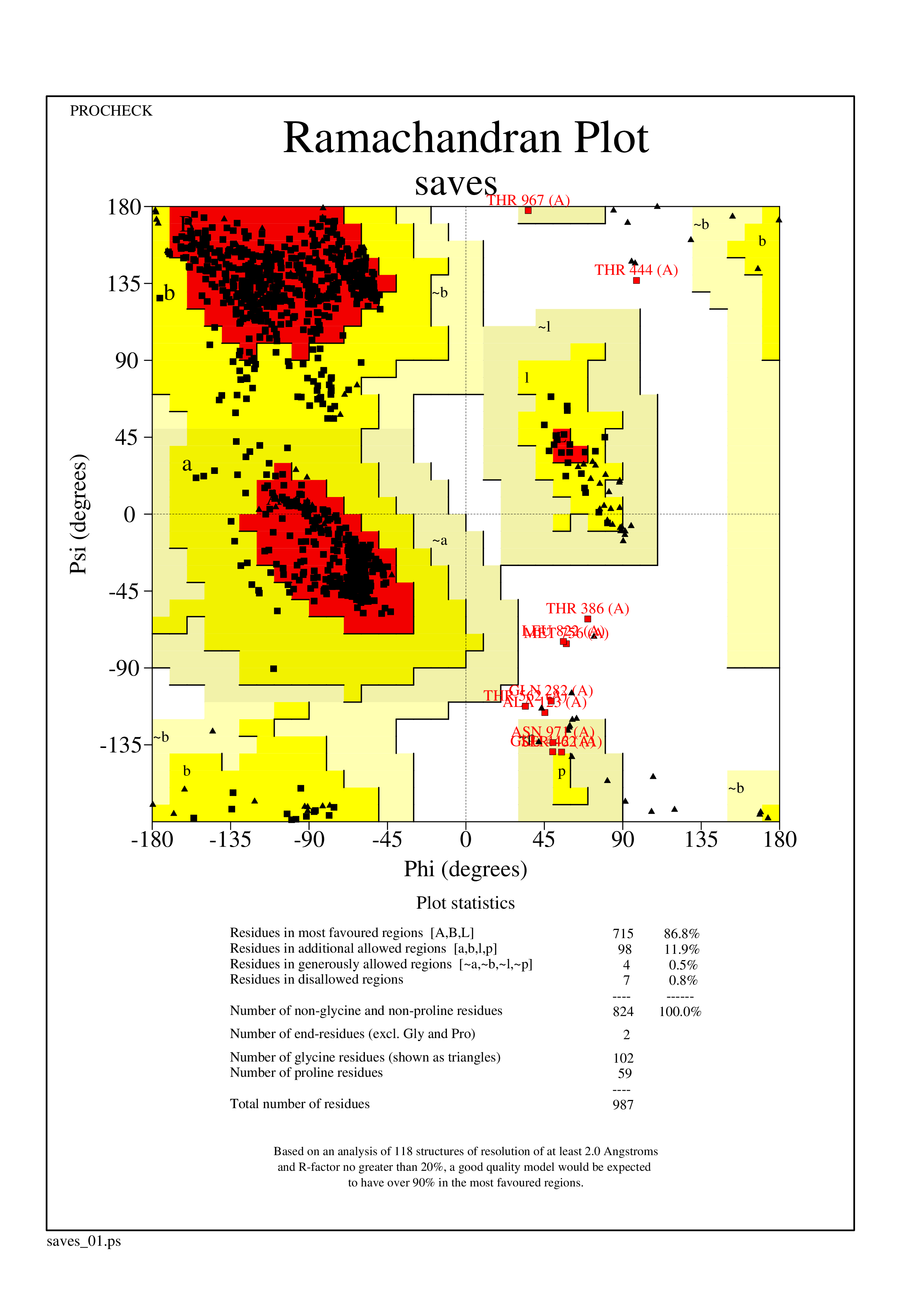

Supplement: S5 File — (ZIP) [file pone.0286428.s005.zip › AorBG2-Ramachandran.png]

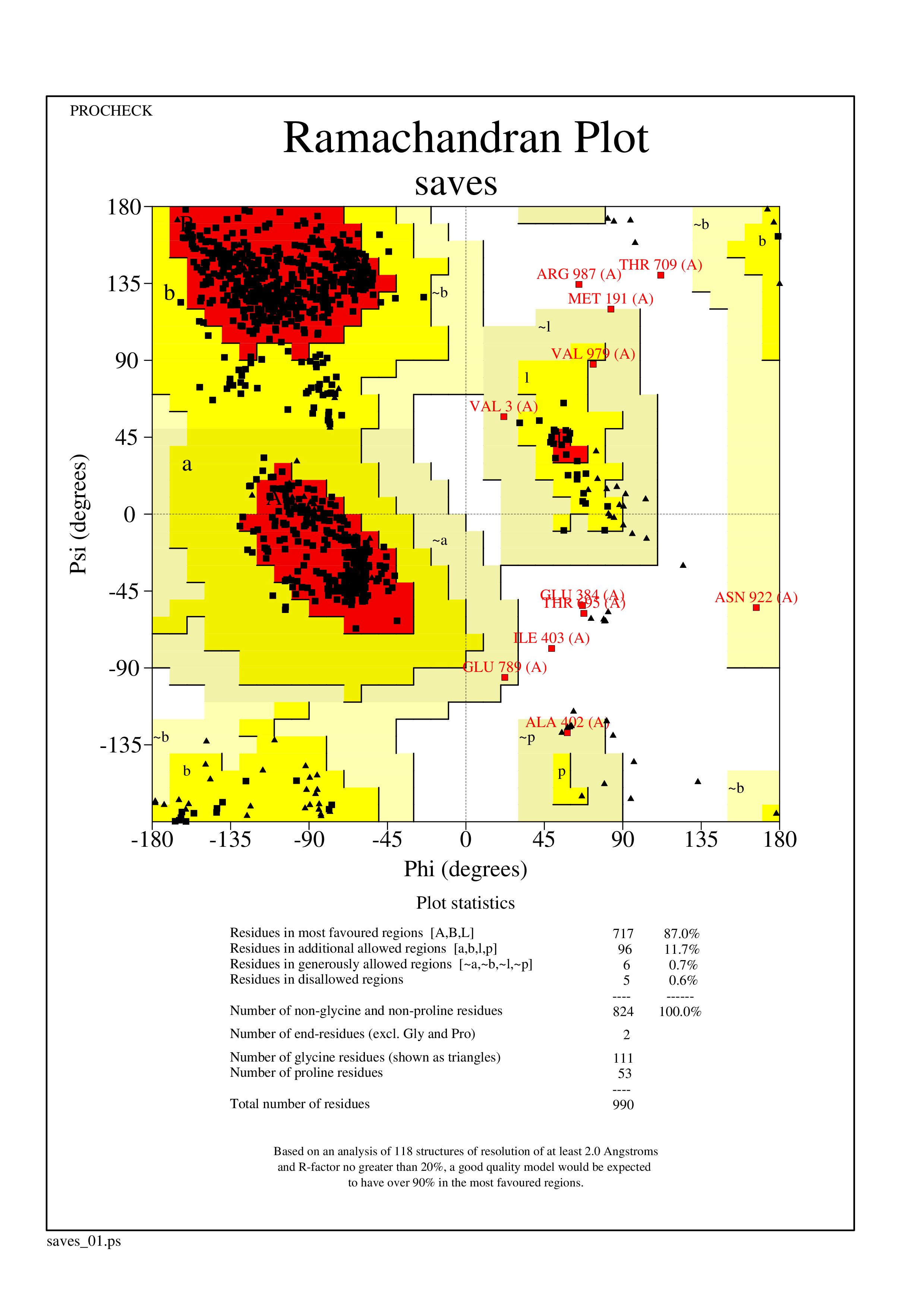

Supplement: S5 File — (ZIP) [file pone.0286428.s005.zip › BciBG3-Ramachandran.png]

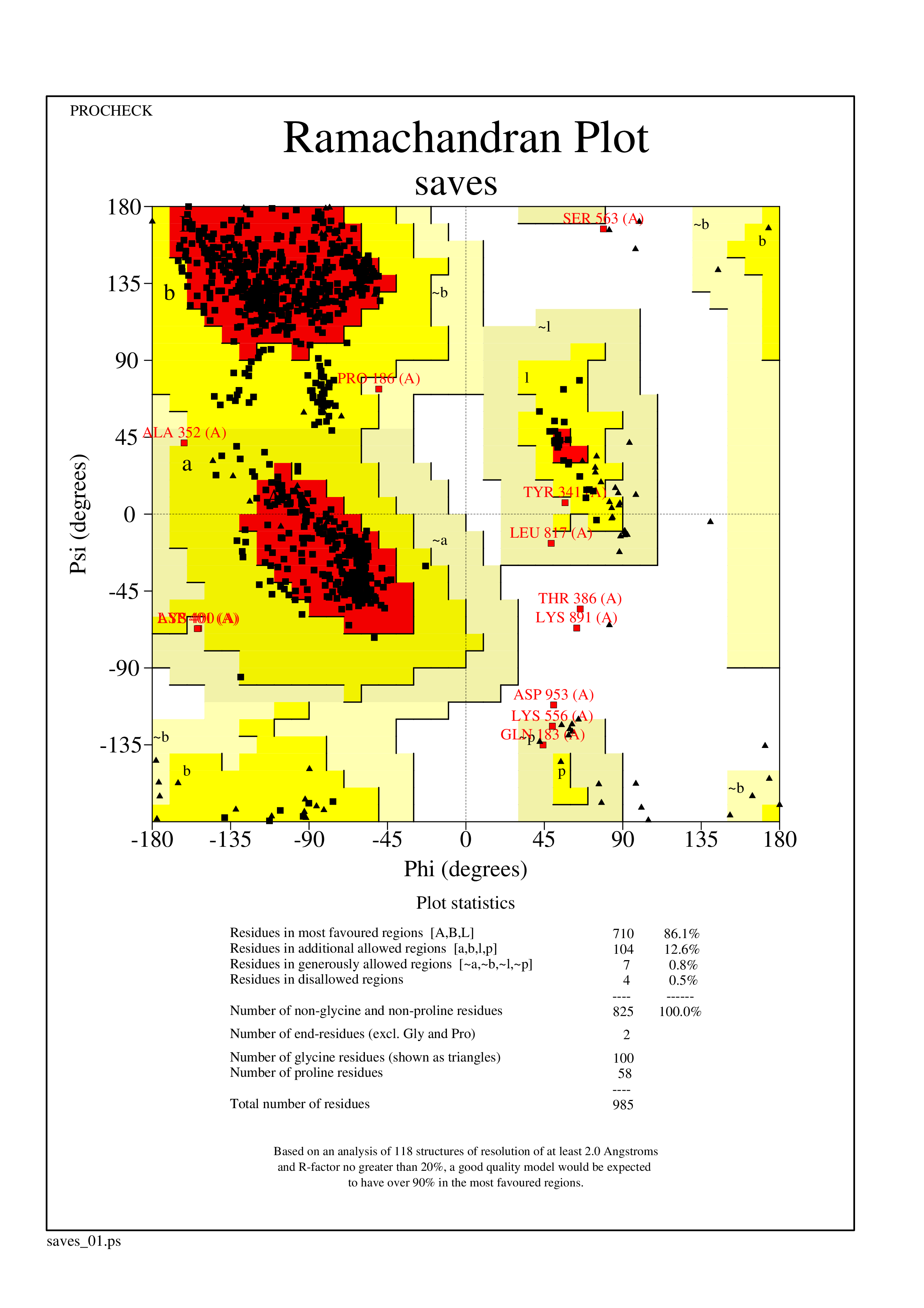

Supplement: S5 File — (ZIP) [file pone.0286428.s005.zip › FfuBG1-Ramachandran.png]

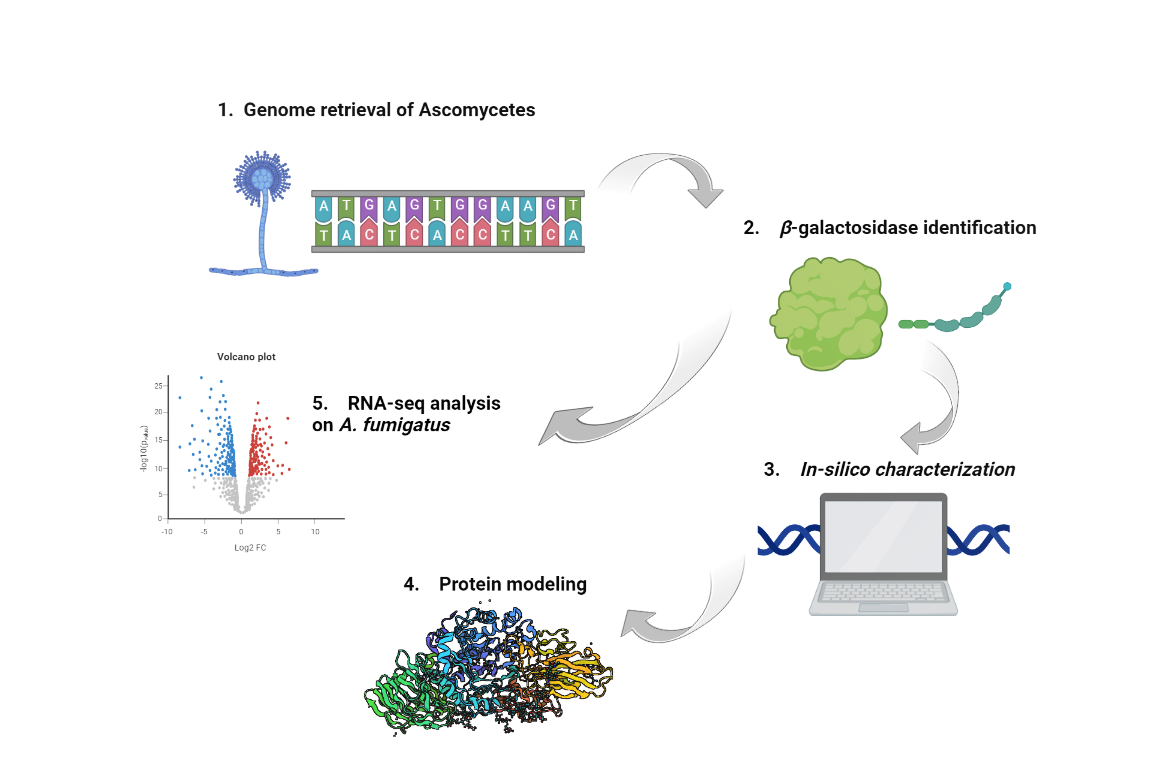

Supplement: S1 Graphical abstract — (TIF) [file pone.0286428.s008.tif]
